# Precise CRISPR-Cas–mediated gene repair with minimal off-target and unintended on-target mutations in human hematopoietic stem cells

**DOI:** 10.1126/sciadv.abm9106

**Published:** 2022-06-03

**Authors:** Ngoc Tung Tran, Eric Danner, Xun Li, Robin Graf, Mikhail Lebedin, Kathrin de la Rosa, Ralf Kühn, Klaus Rajewsky, Van Trung Chu

**Affiliations:** 1 Max Delbrück Center for Molecular Medicine in the Helmholtz Association (MDC), Immune Regulation and Cancer, Berlin, Germany.; 2 Max Delbrück Center for Molecular Medicine in the Helmholtz Association (MDC), Genome Engineering & Disease Models, Berlin, Germany.; 3 Humboldt-Universität zu Berlin, Institute for Biology, Berlin, Germany.; 4 Max Delbrück Center for Molecular Medicine in the Helmholtz Association (MDC), Immune Mechanisms and Human Antibodies, Berlin, Germany.

## Abstract

While CRISPR-Cas9 is key for the development of gene therapy, its potential off-target mutations are still a major concern. Here, we establish a “spacer-nick” gene correction approach that combines the Cas9^D10A^ nickase with a pair of PAM-out sgRNAs at a distance of 200 to 350 bp. In combination with adeno-associated virus (AAV) serotype 6 template delivery, our approach led to efficient HDR in human hematopoietic stem and progenitor cells (HSPCs including long-term HSCs) and T cells, with minimal NHEJ-mediated on-target mutations. Using spacer-nick, we developed an approach to repair disease-causing mutations occurring in the *HBB*, *ELANE*, *IL7R*, and *PRF1* genes. We achieved gene correction efficiencies of 20 to 50% with minimal NHEJ-mediated on-target mutations. On the basis of in-depth off-target assessment, frequent unintended genetic alterations induced by classical CRISPR-Cas9 were significantly reduced or absent in the HSPCs treated with spacer-nick. Thus, the spacer-nick gene correction approach provides improved safety and suitability for gene therapy.

## Teaser

A method for efficient gene repair in human hematopoietic stem and T cells with minimal unwanted genetic alterations is presented.

## INTRODUCTION

The CRISPR-Cas9 system is a powerful tool for gene editing. In this system, single-guide RNAs (sgRNAs) are guiding Cas9 nucleases to the target sequence, which then introduce double-strand breaks (DSBs). In mammalian cells, DSBs are predominantly repaired by the nonhomologous end joining (NHEJ) pathway, causing microinsertions or deletions (indels). If a DNA donor template is provided to a lesser extent, then the homology-directed repair (HDR) pathway is used to precisely replace the mutated DNA sequence (*1*–*4*). In combination with adeno-associated virus (AAV) serotype 6 with single-stranded DNA genome for donor template delivery, preassembled ribonucleoprotein (RNP) complexes of Cas9 nuclease and synthetic sgRNA have led to efficient HDR in human hematopoietic stem and progenitor cells (HSPCs) and T cells (*5*–*7*). The CRISPR-Cas9/AAV6 approach has been used to repair mutations causing several monogenic blood disorders in patient-derived HSPCs (*8*–*10*). Although the CRISPR-Cas9 system succeeded in repairing mutations, its potential off-target effects are still a major concern (*11*–*15*). The CRISPR-Cas9 target specificity has been increased by using truncated sgRNAs (*15*, *16*), extended sgRNAs with two additional G nucleotides at the 5′ end (*17*), and sgRNAs with high specificity (*18*). In addition, off-target effects of Cas9 nuclease were shown to be lower when high-fidelity SpCas9 mutants were used (*19*–*21*). Another strategy to minimize off-target activities is to use the Cas9 nickase, a mutated version of Cas9 in which the RuvC (E. coli protein that is endonuclease) or HNH (histidine-asparagine-histidine motif) nuclease domain has been inactivated by introducing the D10A or H840A mutation, respectively. Cas9 nickase leads to fewer indels than the Cas9 nuclease, because the induced single-stranded breaks (SSBs) are efficiently repaired by the base excision repair pathway (*22*). Previously, Ran *et al*. and Mali *et al*. (*23*, *24*) reported that in combination with a pair of PAM (protospacer-adjacent motif)-out sgRNAs at a distance of 38 to 68 base pairs (bp), the Cas9^D10A^ nickase (referred to as Cas9n hereafter) creates offset double nicks that induce site-specific DSBs, termed as double-nick approach hereafter. The double-nick approach led to efficient HDR and NHEJ events while reducing off-target effects by 50- to 1000-fold (*23*, *24*). The limitation of this strategy is that DSBs are still induced at the target locus, leading to frequent indels and nonsense mutations in the target gene (*25*).

An ideal gene correction approach for therapeutic applications preserves HDR efficiency and, even more important, minimizes adverse effects, such as unintended on- and off-target mutations. To achieve this goal and counteract the introduction of DSBs, we developed an approach, designated as “spacer-nick” below, that combines Cas9n with a pair of PAM-out sgRNAs at a long (>200-bp) spacer distance. Thus, in a first step, Cas9n is guided by the spacer-nick sgRNAs to two target sequences on opposite strands and nicks both DNA strands at an optimal distance to preserve efficient HDR while minimizing NHEJ events. We then combined this system with AAV6-based donor templates to establish gene correction methods to repair mutation hotspots occurring in the *HBB*, *ELANE*, *IL7R*, and *PRF1* genes in primary human HSPCs and T cells. With the use of independent off-target assessment approaches, we confirmed that spacer-nick leads to significantly less unintended on- and off-target mutations than classical CRISPR-Cas9.

## RESULTS

### Gene editing by spacer-nick prevents on-target NHEJ events

**Fig. 1.**
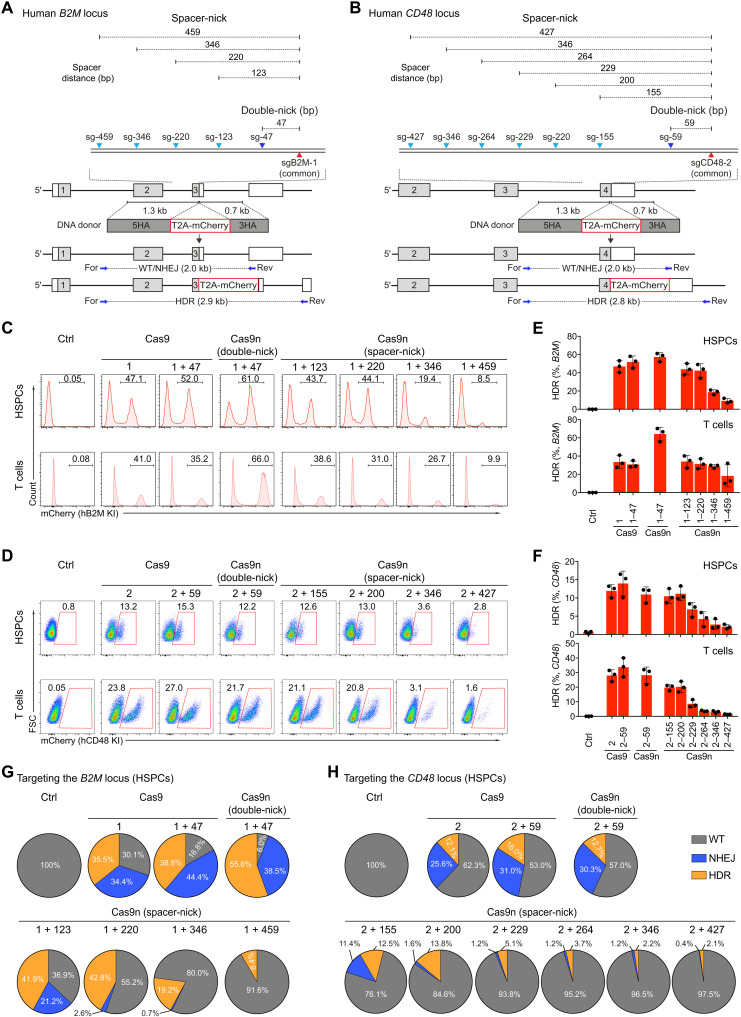
Cas9n and distant sgRNAs lead to HDR and minimal NHEJ in human HSPCs and T cells. (**A** and **B**) Targeting strategy to insert T2A-mCherry into human *B2M* and *CD48* loci. Donor templates with the indicated homology arms (HAs) flanking T2A-mCherry fragments were used. Common sgB2M-1 and sgCD48-2 are indicated as red arrows; PAM-out sgB2M-47 and sgCD48-59 are indicated as dark blue arrows; and spacer-nick sgB2M-123, sgB2M-220, sgB2M-346, sgB2M-459, sgCD48-155, sgCD48-200, sgCD48-229, sgCD48-264, sgCD48-346, and sgCD48-427 are indicated as light blue arrows. Primers used to amplify the wild-type (WT)/NHEJ and HDR sequences are indicated as horizontal blue arrows. (**C** and **D**) Fluorescence-activated cell sorting (FACS) analysis of the percentages of mCherry^+^ HSPCs and T cells targeting the *B2M* and *CD48* loci 3 days after editing. (**E** and **F**) Summary of frequencies of mCherry^+^ HSPCs and T cells after targeting the *B2M* and *CD48* loci. Data are shown as means ± SD based on three independent experiments. (**G** and **H**) Pie charts of the frequencies of WT (gray), NHEJ (blue), and HDR (orange) sequences in the targeted HSPCs treated with the indicated RNPs targeting the *B2M* and *CD48* loci and AAV donor vectors. Data represent the average of three independent experiments.

To quantify HDR and NHEJ efficiencies in human HSPCs and T cells, we designed a targeting reporter system by inserting the coding sequences of a self-cleavage peptide coupled to the fluorescent marker mCherry in-frame into the last exon of the human *B2M* and *CD48* loci. We then designed sgRNAs targeting these genes nearby the STOP codon, termed as sgB2M-1 or sgCD48-2 hereafter, respectively (Fig. 1, A and B). To create “double-nick” sgRNAs, we designed PAM-out sgRNAs with a distance of 47 bp to sgB2M-1 and 59 bp to sgCD48-2. To assess, along the lines of the spacer-nick concept, whether larger sgRNA distances would allow for HDR and prevent NHEJ, we designed several PAM-out sgRNAs whose target sequence was 123 to 459 bp away from that of the first sgRNA (Fig. 1, A and B). On the basis of ICE (Inference of CRISPR Edits) analysis and T7EI assays, all sgRNAs led to efficient target site editing of up to ~80% in HSPCs that were electroporated with the respective sgRNA/Cas9 RNPs (table S1). We then electroporated activated HSPCs and T cells with RNPs containing Cas9 and one or two sgRNAs, or Cas9n with pairs of sgRNAs, and subsequently infected the cells with the respective AAV6 donor templates (fig. S1A). On the basis of the reporter expression (mCherry), quantified by flow cytometry 3 days after targeting, spacer-nick led to HDR efficiencies (~40%) that were similar to the ones observed in the Cas9- or double-nick–based control settings. However, in both cell types and loci, increasing distances between the two sgRNAs correlated with a decreased HDR efficiency (Fig. 1, C to F). To genetically quantify HDR and NHEJ events, we amplified and sequenced the targeted sequences from the genomic DNA of the targeted HSPCs. This analysis uncovered that the double-nick and Cas9 approaches led to efficient NHEJ of ~40% (*B2M*) and ~30% (*CD48*), whereas the spacer-nick approach with a long (>200-bp) spacer distance almost exclusively led to HDR events (Fig. 1, G and H, and fig. S1B). Consistent with the reporter system, similar HDR rates were observed in spacer-nick–, Cas9-, and double-nick–treated cells, and increasing spacer distances of spacer-nick sgRNAs resulted in a significant reduction of HDR in HSPCs and T cells (Fig. 1, C to H, and fig. S1, C and D). Thus, a spacer distance of 200 to 350 bp in the spacer-nick system minimizes on-target NHEJ-dependent indel frequencies and mediates efficient HDR in human HSPCs and T cells. Similar observations have been made in human induced pluripotent stem (iPS) cells and cell lines using in trans and tandem paired nicking approaches that combine Cas9n with sgRNAs that induce DNA nicks at both the target sequence and double-stranded DNA donor plasmids. The in trans paired nicking approach induces a DNA nick at the target sequence and one or two nicks into the DNA donor plasmid. In the tandem paired nicking approach, two nicks are introduced into one of the strands of the target gene and the DNA donor plasmid, respectively (*26*–*28*). To compare these methods with spacer-nick in human HSPCs, we introduced nicks in the double-stranded region of self-complementary (sc) AAV vectors and used these as donor templates for the T2A-mCherry insertion into the *B2M* locus. Unexpectedly, both the in trans and tandem paired nicking methods led to very low HDR rates (<1%) in human HSPCs in contrast to the ~30-fold higher HDR rates achieved with spacer-nick (figs. S2B and S3). These data suggest that the spacer-nick method is unique in leading to efficient HDR and low on-target NHEJ events in human HSPCs.

### Application of spacer-nick on mutation hotspots in HSPCs and T cells

**Fig. 2.**
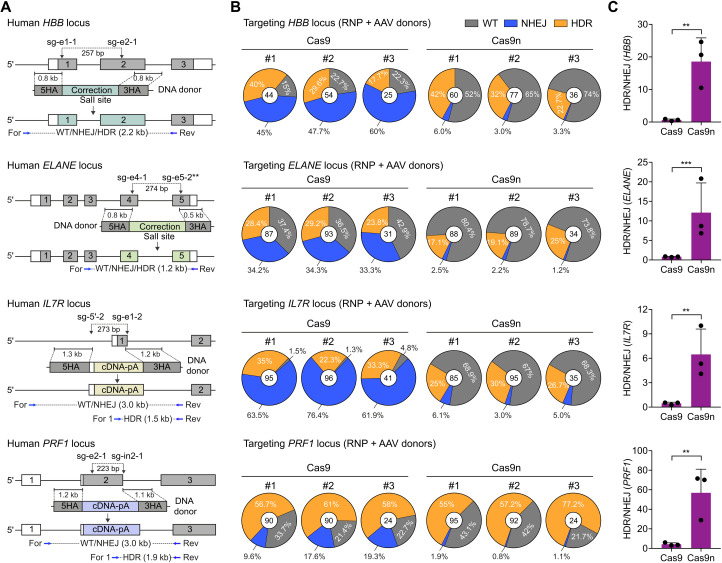
Spacer-nick–based gene correction of mutation hotspots at the *HBB*, *ELANE*, *IL7R*, and *PRF1* loci in human HSPCs. (**A**) Strategy to repair mutations occurring in the exons 1 and 2 of *HBB*, in the exons 4 and 5 of *ELANE*, or in coding and intronic sequences of *IL7R* and *PRF1* genes. A pair of spacer-nick sgRNAs targeting the *HBB*, *ELANE*, *IL7R*, and *PRF1* loci; and their spacer distances are indicated. Donor templates with indicated length of HAs including repair sequence and a Sal I recognition site in the cases of *HBB* and *ELANE* or codon-modified cDNA and poly-A sequences in the cases of *IL7R* and *PRF1* are depicted. Primers used to amplify the targeted alleles are shown. (**B**) Pie charts showing percentages of WT (gray), NHEJ (blue), and HDR (orange) sequences at the targeted *HBB*, *ELANE*, *IL7R*, and *PRF1* loci in the HSPCs treated with either sgRNAs/Cas9 (Cas9) or spacer-nick (Cas9n) RNPs and AAV donor vectors. The number of analyzed reads is indicated in center of the pie charts. Data are represented from three independent experiments. (**C**) Bar graphs showing the ratio of HDR:NHEJ events shown in (B) at the indicated loci in human HSPCs. Data are shown as means ± SD and based on three independent experiments. ***P < 0.001; **P < 0.01 (Mann-Whitney test).

To test the spacer-nick system on clinically relevant loci in primary immune cells, we focused on a set of known mutations in the *HBB*, *ELANE*, *IL7R*, and *PRF1* genes that are known to cause beta thalassemia, severe congenital neutropenia, severe combined immune deficiency, and familial hemophagocytic lymphohistiocytosis, respectively. Approximately 80 and 60% of the known mutations are located in exons 1 and 2 of the *HBB* and in exons 4 and 5 of the *ELANE*, respectively (*29*, *30*). In contrast, the disease-causing mutations in the case of *IL7R* and *PRF1* are randomly distributed in coding and intronic sequences (*31*, *32*). To correct the *HBB* and *ELANE* mutation hotspots, we generated DNA donor templates containing silent mutations (to reduce homology) and a diagnostic Sal I restriction enzyme site. To repair the *IL7R* and *PRF1* mutations, we generated DNA donor templates including homology arms (HAs) flanking codon-modified cDNA and polyadenylation (poly-A) sequences. Next, we designed and tested pools of sgRNAs flanking the mutation hotspots in the *HBB*, *ELANE*, *IL7R*, and *PRF1* loci (table S1). The most efficient pairs of sgRNAs, with a distance of 200 to 350 bp, were then chosen for a “universal” spacer-nick gene correction approach in HSPCs and T cells (Fig. 2A). As controls, Cas9 nucleases were combined with the same two sgRNAs (*HBB*, *IL7R*, and *PRF1*) or one sgRNA (*ELANE*). A first analysis based on Sal I–mediated restriction fragment length polymorphism (RFLP) (*HBB* and *ELANE*) and correct integration polymerase chain reaction (PCR) (*IL7R* and *PRF1*) indicated gene correction efficiencies of 20 to 50% 3 days after targeting, similar to the HDR efficiency in the Cas9-treated cells (figs. S4 and S5). To precisely quantify NHEJ and HDR events on the target sites, we sequenced the target PCR products amplified from genomic DNA of the targeted HSPCs and T cells. Consistent with the B2M (beta-2-microglobulin)/CD48 (CD48 antigen) findings, the spacer-nick and CRISPR-Cas9 systems led to efficient HDR at all targeted loci, while NHEJ events were again significantly lower in spacer-nick–treated cells (Fig. 2B and fig. S5). As a result, the HDR:NHEJ ratio at all targeted loci was significantly higher with the spacer-nick system (Fig. 2C and figs. S5 and S6). We achieved similar results with additional pairs of spacer-nick sgRNAs (table S2). Thus, the spacer-nick system allows to repair mutations in the *HBB*, *ELANE*, *IL7R*, and *PRF1* genes in human HSPCs and T cells with minimal unintended on-target mutations.

### Spacer-nick leads to precise and efficient HDR with minimal NHEJ in long-term HSCs

**Fig. 3.**
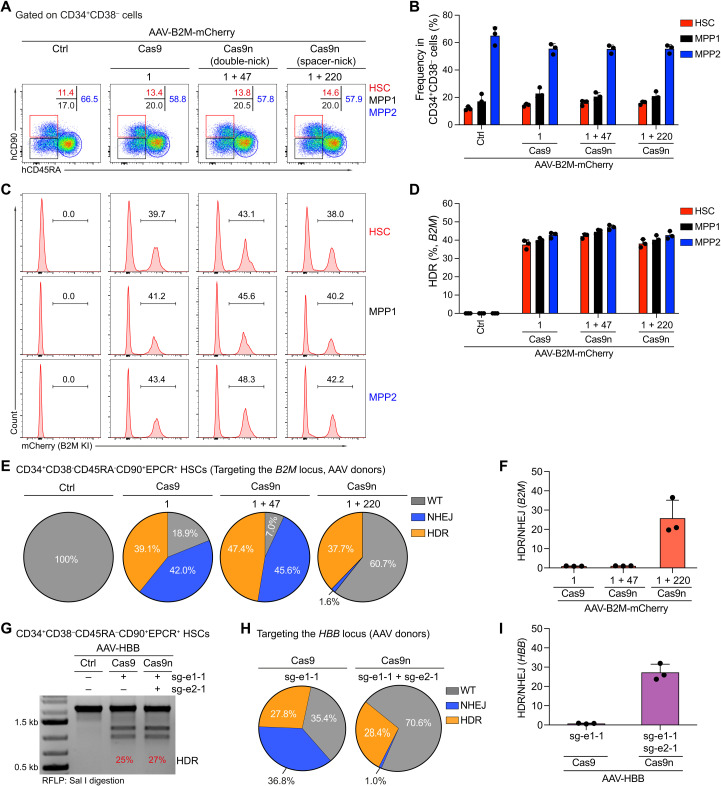
Spacer-nick leads to precise and efficient HDR with minimal NHEJ events in long-term HSCs. (**A**) FACS analysis, pregated on CD34^+^CD38^−^ cells, showing frequencies of HSC (CD90^+^CD45RA^−^, red), MPP1 (CD90^−^CD54RA^−^, black), and MPP2 (CD90^−^CD45RA^+^, blue) subsets 3 days after targeting the *B2M* locus in human CD34^+^ cells treated with control (Ctrl; no RNPs), Cas9, double-nick, or spacer-nick RNPs and AAV-B2M-mCherry vectors. (**B**) Summary of frequencies of HSC, MPP1, and MPP2 subsets in (A) based on three independent experiments. (**C**) FACS analysis showing percentages of mCherry^+^ cells within HSC, MPP1, and MPP2 subsets. (**D**) Bar graph showing frequencies of mCherry^+^ HSC, MPP1, and MPP2 cells in (C). Data are shown as means ± SD based on three independent experiments. (**E**) Pie charts of frequencies of WT (gray), NHEJ (blue), and HDR (orange) sequences at the targeted *B2M* locus in the sorted long-term HSCs (CD34^+^CD38^−^CD45RA^−^CD90^+^EPCR^+^) 3 days after targeting. Summary of the ratio of HDR:NHEJ events at the targeted *B2M* locus in long-term HSCs treated as indicated. Data are shown as means ± SD and based on three independent experiments. (**G**) Sal I–mediated RFLP assay showing the gene correction efficiency of the targeted *HBB* locus in long-term HSCs treated as indicated. (**H**) Pie charts showing frequencies of WT (gray), NHEJ (blue), and HDR (orange) sequences at the targeted *HBB* locus in long-term HSCs. (**I**) Bar graph representing the ratio of HDR:NHEJ events at the targeted *HBB* locus in long-term HSCs. Data are shown as means ± SD and based on three independent experiments.

To address whether spacer-nick leads to efficient HDR and minimal on-target NHEJ events in human long-term HSCs, we targeted the *B2M* and *HBB* loci in human CD34^+^ cells (Figs. 1A and 2A). As controls, Cas9 nucleases were combined with one sgRNA (*B2M* and *HBB*) or two sgRNA-based double-nick approach (*B2M*). Long-term engrafting HSCs are enriched in the CD34^+^CD38^−^CD45RA^−^CD90^+^EPCR^+^ population, whereas multipotent progenitor (MPP) subsets were defined as CD34^+^CD38^−^CD45RA^−^CD90^−^ (MPP1) and CD34^+^CD38^−^CD45RA^+^CD90^−^ (MPP2), respectively (*33*–*36*). We detected similar percentages of the HSC, MPP1, and MPP2 subsets in controls (Ctrl, no RNPs), Cas9-, double-nick–, or spacer-nick–treated cells 3 days after targeting the *B2M* locus (Fig. 3, A and B, and fig. S7). On the basis of the mCherry expression, Cas9, double-nick, and spacer-nick approaches led to similar HDR efficiencies (~40%) within the HSC, MPP1, and MPP2 subsets (Fig. 3, C and D). To genetically quantify HDR and NHEJ events in long-term HSCs, we sorted the *B2M*- and *HBB*-targeted CD34^+^CD38^−^CD45RA^−^CD90^+^EPCR^+^ HSCs and analyzed the loci by Sanger sequencing. Consistent with the mCherry expression data, we detected similar HDR rates in Cas9-, double-nick (*B2M*)–, and spacer-nick–treated cells. However, the Cas9 and double-nick approaches led to NHEJ frequencies of ~42 (*B2M*) and 36% (*HBB*), whereas the spacer-nick gene correction approach led to a more than 20-fold decrease in NHEJ frequencies (~1.6%) (Fig. 3, E to I, and fig. S7D). Thus, the spacer-nick system led to precise and efficient HDR with minimal unwanted on-target mutations in human long-term engrafting HSCs.

### Genome-wide reduction of off-target mutations in spacer-nick

**Fig. 4.**
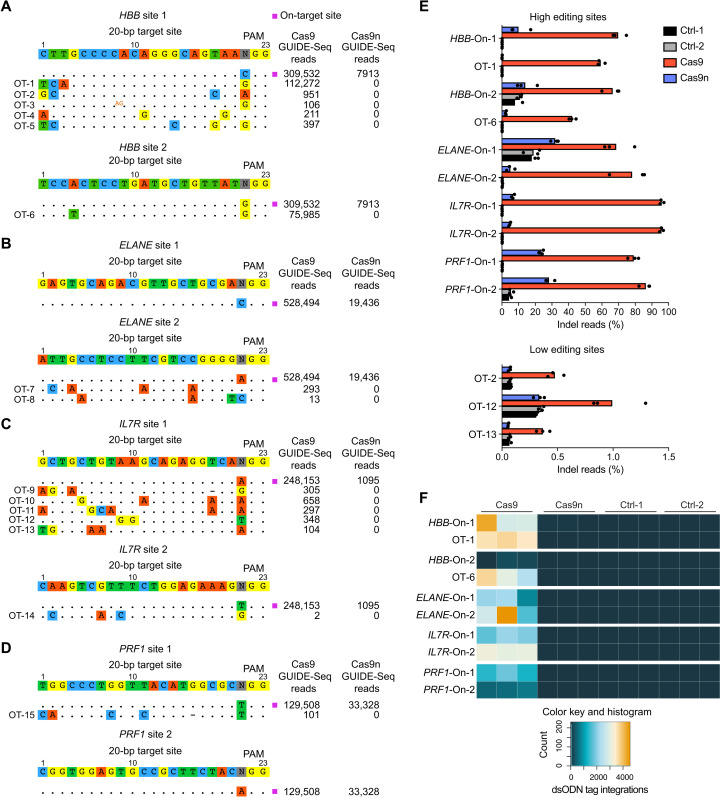
Reduced on-target DSBs and off-target editing in the HSPCs treated with spacer-nick. (**A** to **D**) GUIDE-seq results of the indicated sgRNAs/loci based on HSPCs treated with either sgRNAs/Cas9 (Cas9) or spacer-nick (Cas9n) RNPs. The detected on-target (magenta square) and off-target (OT) sequences are shown with the respective mismatches to the protospacer sequence. Insertions are indicated as superscript (orange). (**E**) Frequencies of indel reads quantified by amplicon-sequencing at on- and off-target sites in untreated HSPCs (Ctrl-1), HSPCs treated with dsODN only (Ctrl-2), sgRNAs/Cas9 (Cas9), or sgRNAs/Cas9n (Cas9n) RNPs. Data are shown as means ± SD from three independent experiments. (**F**) Heatmap of the number of dsODN tag integrations, detected by amplicon sequencing, on- and off-target sites in the HSPCs treated as indicated. Data are based on three independent experiments.

The low frequency of NHEJ events in cells treated with spacer-nick suggested a beneficial effect with respect to off-target editing. To quantify genome-wide off-target mutations, we applied a modified version of GUIDE (Genome-wide, Unbiased Identification of DSBs Enabled by Sequencing)-seq (sequencing) on the HSPCs treated as shown in Fig. 2, but without AAV6 donor vectors (fig. S8). GUIDE-seq is based on the integration of a blunt-end double-stranded oligodeoxynucleotide (dsODN) into the nuclease-introduced DSBs (*15*). PCR amplification with primers that annealed to each of the dsODN strands and Illumina adapters allowed next-generation sequencing and analysis through our pipeline based on the original GUIDE-seq software (fig. S9) (*15*). Ten days after targeting, frequent dsODN integrations in on-target sites and top off-target sites predicted by CRISPOR (*37*) and CrispRGold (*38*) were detected in the Cas9-treated HSPCs. In contrast, Cas9n-treated HSPCs showed a significant and up to ~260-fold reduction in detected on-target reads. Moreover, not a single dsODN integration site was detected in the off-targets in Cas9n-treated cells (Fig. 4, A to D). The GUIDE-seq method is relevant to measure DSB-mediated off-target mutations; however, its limitation is its inability to detect mutations produced by SSBs that may prevent integrations of the blunt-end dsODNs. The nicks introduced by the Cas9 nickases may still lead to point mutations in off-target sites (*39*). To address this point, we performed amplicon deep sequencing for on-target and potential high-risk off-target sites, identified by GUIDE-seq, on the genomic DNA obtained in the GUIDE-seq experiments (fig. S8). The amplicon-seq analysis revealed that Cas9n-treated HSPCs showed significantly less indel events at on-target sites, compared to the Cas9-treated cells. In addition, multiple off-target sites were extensively modified by Cas9, whereas Cas9n led to indel rates that were close to background levels (untreated cells or dsODN-treated cells) (Fig. 4E and fig. S10). Consistent with the GUIDE-seq data, dsODN tag integrations detected by amplicon sequencing were exclusively present in the cells treated with Cas9 nucleases (Fig. 4F and fig. S10). Thus, the spacer-nick system significantly reduces unwanted on- and off-target mutations.

### General reduction of unwanted genetic alterations in spacer-nick

**Fig. 5.**
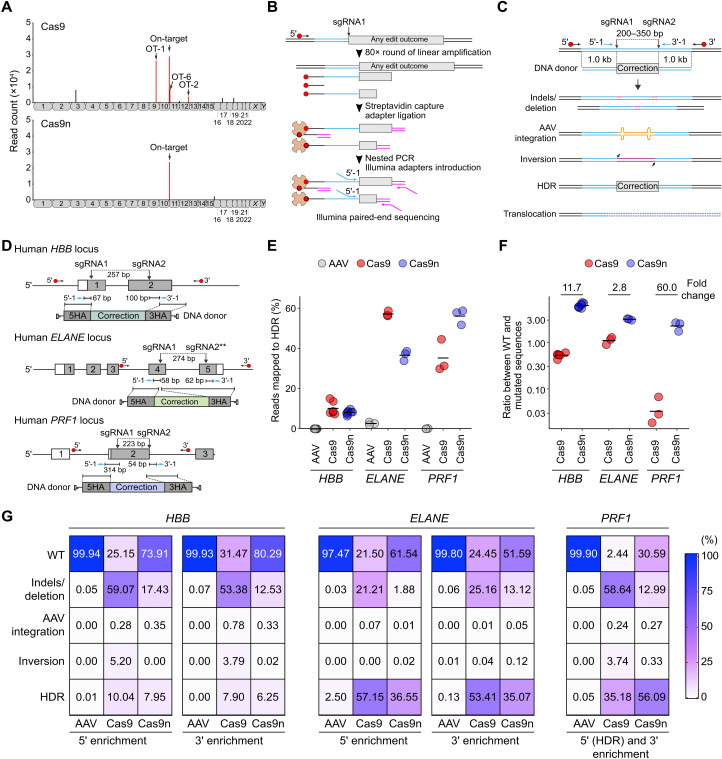
Analysis of AAV integrations and complex genetic alterations by AAV-seq and LAM-HTGTS. (**A**) AAV integration sites identified and mapped on human chromosomes of *HBB*-targeted HSPCs treated with sgRNAs/Cas9 (Cas9) or sgRNAs/Cas9n (Cas9n) RNPs and AAV donor vectors. The top hits of AAV integrations are indicated in red, including the on-target site and three high-risk off-target sites (OT-1, OT-2, and OT-6), as identified by GUIDE-seq. Random AAV integrations are shown in black. (**B**) Scheme of LAM-HTGTS. Red circles depict biotin, and orange counterparts depict streptavidin. (**C**) Scheme of the spacer-nick gene repair approach (top) and all the possible genetic outcomes including small indels/and deletion, AAV integration, inversion, HDR events, and translocations. LAM-PCR was performed using external biotinylated primers annealing outside of the 5′ or 3′ HAs and nested primers nearby the first cleavage site (5′-1 or 3′-1). (**D**) Schematic representation of the targeted loci analyzed by LAM-HTGTS. Two asterisks indicate that this sgRNA was not combined with Cas9. (**E**) HDR efficiencies detected by LAM-HTGTS at the targeted loci. (**F**) Ratio of WT:mutated sequences in the HSPCs treated as indicated. Data are shown as means ± SD from at least three independent experiments. (**G**) Heatmaps of the frequencies of all outcomes depicted for both 5′ and 3′ enrichments at the targeted *HBB* and *ELANE* loci or combined enrichment (5′ and 3′) at the targeted *PRF1*.

In addition to indels at on- and off-target sites, gene targeting may lead to vector integrations or translocations. Integrations of AAV vectors into the host genome are known to occur (*40*, *41*). To address whether these complex genetic events occur in the spacer-nick system, we focused on the *HBB*-corrected HSPCs. To rule out AAV integrations, we performed a modified version of AAV-seq, in which the integrated AAV is used as primer template to identify the integration sites (fig. S8) (*42*). The AAV-seq data were processed similar as the GUIDE-seq data (fig. S9). This analysis revealed that low levels of AAV integrations were detected in control cells that were only treated with AAV donor vectors 18 days before analysis (fig. S11B). As expected, significantly higher numbers of AAV integrations were detected at the on-target site in Cas9- and Cas9n-treated cells. In contrast, the frequent AAV integration sites at the off-target sites OT-1 and OT-2 under Cas9 conditions were not detected in the Cas9n-treated cells, and OT-6, proximal to the on-target site, showed significantly lower levels of AAV integrations (Fig. 5A and fig. S11). A limitation of the AAV-seq method is, however, that it is not quantitative with respect to the frequencies of AAV integrations at on-target sites because of the PCR step involved.

To comprehensively map all possible gene-editing outcomes induced by the various gene-editing approaches at the *HBB*, *ELANE*, and *PRF1* loci in human HSPCs, we used the linear amplification-mediated high-throughput genome-wide translocation sequencing (LAM-HTGTS) method (Fig. 5B and fig. S9) (*12*). This method is based on linear amplification and leads to a 5′- and 3′-based sequencing of the target locus, allowing for a quantification of indels/and deletion, AAV integrations, inversions, gene repair (HDR), and translocations. To exclude any contamination by remaining AAV donor vectors, we used primers outside of HAs for the first round of linear amplification (Fig. 5, C and D). No translocations were detected in all conditions. Consistent with the previous findings, gene correction efficiencies of ~10 to 60% were detected at the targeted loci (Fig. 5, E and G). Moreover, the ratio of wild-type versus mutated sequences was significantly higher in Cas9n-treated cells (~3- to 60-fold), confirming the reduced NHEJ rates detected by Sanger sequencing (Figs. 2 and 5, F and G). Overall, these data confirm that the spacer-nick gene correction approach retains high HDR efficiency, while unintended genetic alterations are significantly reduced.

## DISCUSSION

CRISPR-Cas9–based gene correction holds great potential for gene therapy to cure monogenic disorders. Despite intensive development and improvement, the safety of the system remains a major clinical concern. With spacer-nick, we established a precise and safe gene correction approach that is based on Cas9n and a pair of sgRNAs at a distance of 200 to 350 bp. In combination with AAV6 donor templates, this system led to efficient gene repair with minimal unintended on- and off-target editing in human HSPCs and T cells.

Consistent with previous reports, the classical CRISPR-Cas9 system induced frequent off-target mutations and unwanted genetic alterations in our experiments (*2*, *11*–*15*, *17*). Our Cas9n-based results are in agreement with previous studies, in which Cas9 nickases with a pair of short-distance (38 to 68 bp) PAM-out sgRNAs strongly reduced off-target effects (*23*, *24*). Although this latter double-nick approach induced efficient HDR and prevented off-target editing, the two juxtaposed SSBs may still be processed into DSBs that are predominantly repaired by the NHEJ pathway, leading to unwanted mutations and potentially dysfunctional gene products (*25*). Such unwanted on-target indels may produce activating/or dominant mutations in oncogenes, such as *NOTCH1*, or truncated dominant negative gene products, and thereby cause tumorigenesis or abnormal hematopoiesis. Thus, on-target indels should be avoided during gene targeting.

To avoid on-target indels, a safe gene correction approach for therapeutic applications needs to minimize unintended on-target DSBs while retaining HDR rates. Although the in trans and tandem paired nicking approaches have achieved this in human iPS cells and cell lines (*26*–*28*), their efficiency in human HSPCs appears to be low based on our results, potentially due to a distinct DNA nick repair mechanism active in these cells. By using a pair of distant sgRNAs in spacer-nick (200 to 350 bp), we significantly reduced the on-target DSBs and indels that we observed in the double-nick approach. Because of their distance, these SSBs appear to be predominantly repaired by the base excision repair pathway, leading to a reduction of DSB-mediated on-target indels (*22*). In the presence of AAV6 donor templates, the distant SSBs may be repaired by an alternative HDR pathway (*43*). The precise mechanism of the DNA repair pathway active in spacer-nick remains to be elucidated.

Finding a potent pair of PAM-out sgRNAs with a spacer distance of 200 to 350 bp is crucial to achieve efficient HDR while minimizing NHEJ events using the spacer-nick approach. To find the best pairs, we designed pools of PAM-out sgRNAs flanking two potential target sites separated by 200 to 350 bp and chose the best sgRNA pairs with high and similar editing efficiencies (>80%). In combination with a pair of PAM-out sgRNAs, Cas9n creates two distant nicks with a spacer distance of 200 to 350 bp on opposite strands of the targeted DNA. The sequence between the two nicks may serve as a mini-HA (termed as mini–homologous sequence) that potentially leads to partial homologous recombination in the presence of DNA donor templates. To reduce the length of this mini–homologous sequence and avoid partial homologous recombination, we generated DNA donor templates harboring 5′ and 3′ HAs of at least 800 bp outside of each nick site. Moreover, we modified the coding sequences inside of the mini–homologous sequences by introducing silent mutations (*HBB* and *ELANE*) or by partially ablating these sequences (*IL7R* and *PRF1*) in the DNA donor templates.

As a proof of principle, we used spacer-nick to develop universal gene correction systems that would allow to repair hotspots of mutations occurring in the *HBB*, *ELANE*, *IL7R*, and *PRF1* genes. At these loci, we achieved 20 to 50% of gene correction efficiencies in human HSPCs and T cells. We provide a standardized workflow to assess gene correction efficiency and off-target activities after gene editing in a clinical setup. These data show that the spacer-nick system is a safe and efficient gene-editing approach that may be used in therapies of beta thalassemia, severe congenital neutropenia, severe combined immune deficiency, familial hemophagocytic lymphohistiocytosis, and other monogenic blood disorders.

## MATERIALS AND METHODS

### Isolation and culture human HSPCs and T cells

Human CD34^+^ HSPCs were isolated and cultured as previously described (*44*). Human CD3^+^ T cells were isolated from the peripheral blood of healthy donors using Pan T cell isolation kit according to the manufacturer’s protocol (Miltenyi Biotec). A total of 1 × 10^6^ CD3^+^ T cells were stimulated with anti-human CD3/CD28 Dynabeads (Thermo Fisher Scientific) at a ratio of 1:1. Three days later, the beads were removed, and the activated T cells were further cultured in 1 ml of RPMI 1640 (Gibco) supplied with 10% fetal calf serum, 1× GlutaMAX, and human interleukin-2 (IL-2) (100 U/ml; PeproTech). Human granulocyte colony-stimulating factor–mobilized peripheral blood cells were obtained from the Stem Cell Core Facility at Charité Hospital as an excessive apheresis product. Peripheral blood from healthy donors was obtained from the Charité blood bank. The study protocol has been reviewed and approved by the Institutional Review Board at Charité Hospital. Informed consent was obtained from all donors.

### AAV donor template cloning and AAV production

To achieve efficient HDR rates using the spacer-nick system, we generated DNA donor templates carrying 5′ and 3′ HAs of at least 800 bp outside of each nick site. To generate pAAV-B2M or CD48-T2A-mCherry donor vectors, the left and right HAs were amplified from human genomic DNA and cloned into Xho I/Eco RI and Asi SI/Kpn I sites of the pTV-T2A-mCherry vectors, respectively. The Not I–flanked B2M or CD48-T2A-mCherry fragments were cloned into the pAAV vector (Cell Biolabs). To generate pscAAV-B2M-T2A-mCherry without or with sgRNA-targeted sequences, Avr II/Spe l flanked B2M-T2A-mCherry fragments were cloned into the pscAAV vectors (Cell Biolabs), and sequentially, sgRNA-targeted sequences were cloned into pscAAV-B2M-T2A-mCherry vectors. To generate pAAV-HBB (Hemoglobin Subunit Beta), pAAV-IL7R (Interleukin 7 receptor), or pAAV–PRF1 (prolactin-releasing factor 1) donor vector, HBB (exon 1 and 2), IL-7 receptor (IL7R) (modified cDNA), or PRF1 (Perforin 1) (modified cDNA) donor template including HAs, silent mutations, a Sal I recognition site, codon-modified cDNA, and poly-A sequences was synthesized by GeneArt Gene Synthesis (Thermo Fisher Scientific) and subsequently cloned into pAAV vector (Cell Biolabs). The pAAV-ELANE (neutrophil elastase) donor vector was previously generated, and human embryonic kidney 293T culture and AAV production were described in detail in our previous publication (*44*).

### sgRNA, dsODN, and RNP electroporation and AAV infection

Pools of PAM-out sgRNAs flanking two target sites with a distance of 123 to 459 bp were designed using the CrispRGold program (*38*) and purchased from Synthego or Integrated DNA Technologies (IDT) (table S1). In addition, we also used previously published sgRNAs targeting the exon 1 of the *HBB* (sg-e1-1 and sg-e1-2) (*5*, *45*) and the exon 4 of the *ELANE* (sg-e4-1) (*44*) genes. SpCas9, SpCas9n (Cas9^D10A^), and dsODN (34 nucleotides) were purchased from IDT. To generate the sgRNA complexes, crRNA (CRISPR RNA) (100 pmol, 1.2 μg) and tracrRNA (trans-activating crRNA) (100 pmol, 1.2 μg) were mixed at a 1:1 ratio, incubated at 95°C for 5 min, and ramped down to room temperature. To generate the RNP complexes, Cas9 or Cas9n (50 pmol, 8.2 μg) was mixed with sgRNAs (100 pmol, 3.3 μg) at a 1:2 molarity ratio and incubated at 25°C for 10 min. A total of 2 × 10^5^ human CD34^+^ HSPCs and T cells were suspended into 20 μl of P3 electroporation buffer (Lonza) containing RNPs. After electroporation with RNPs, human HSPCs (DZ-100) and T cells (EH-100) were transferred to a prewarmed medium supplied with cytokines and placed into an incubator at 37°C and 5% CO_2_. Fifteen to 30 min later, the rAAV6 donor particles were added to electroporated cells at a multiplicity of infection (MOI) 1 × 10^5^ GC (genome copy) per cell for HSPCs and a MOI 1 × 10^6^ GC per cell for human T cells. For GUIDE-seq experiments, HSPCs were electroporated with RNPs and 25 pmol of duplexed dsODN. The medium was changed the next day. The targeted cells were harvested at different time points. The dead cells were removed by using a dead cell removal kit according to the manufacturer’s protocol (Miltenyi Biotec). The living cells were analyzed or harvested for genomic DNA extraction for further analysis.

### Antibodies and FACS analysis

Three days after targeting, the edited HSPC and T cells were collected, washed with fluorescence-activated cell sorting (FACS) buffer (phosphate-buffered saline/1% bovine serum albumin), and analyzed by BD LSRFortessa. The data were analyzed using FlowJo. The targeted CD34^+^ HSPCs were stained with the following antibodies: APC (allophycocyanin) anti-human CD34 [Research Resource Identifiers (RRID): AB_2228972], PE/Cy7 anti-human CD38 (RRID: AB_2072782), APC/Cy7 anti-human CD45RA (RRID: AB_10708880), Brilliant Violet 605 anti-human CD90 (Thy1) (RRID: AB_2562281), and PE (phycoerythrin) anti-human CD201 (EPCR) (RRID: AB_10900806). All antibodies were purchased from BioLegend. The targeted CD34^+^CD38^−^CD45RA^−^CD90^+^EPCR^+^ HSCs were sorted using Aria sorters (BD FACSDiva), and genomic DNA was then extracted for further analysis.

### PCR, T7EI, and RFLP assays and Sanger sequencing

Genomic DNA from the targeted HSPCs and T cells was extracted using the QuickExtract DNA extraction kit (Epicentre) following the manufacturer’s protocol. To assess gene editing efficiencies of sgRNAs, the targeted sequences were amplified from genomic DNA by PCR (30 cycles) using the Herculase II Fusion DNA Polymerase (Agilent Technology) with gene-specific primers (table S3). PCR products were cleaned using AMPure XP beads (Beckman Coulter). The purified PCR products were subjected to T7EI assay as described previously (*4*). For ICE analysis, the purified PCR products were sequenced using Sanger sequencing and then analyzed by ICE tool (Synthego). Percentage of indels was calculated on the basis of the decomposition algorithm and represented as ICE score (*46*). For correct integration of PCR and RFLP assays, the targeted fragments were amplified using the KOD (*P. kodakaraensis*) Hot Start DNA polymerase (Millipore) with gene-specific primers (table S3). PCR products were purified using AMPure XP beads (Beckman Coulter) and digested with Sal I restriction enzyme for 1 hour at 37°C. Cleaved DNA fragments were separated on 1.2% agarose gel. DNA concentration of each band was quantified using ImageJ software (National Institutes of Health). Percentages of indels and HDR were calculated as described (*1*). For quantifying HDR and NHEJ events on the targeted loci, the purified PCR products were cloned into the sequencing plasmids using CloneJET PCR cloning kit (Thermo Fisher Scientific) following the manufacturer’s protocol. Colonies were picked into 96-well LB agar plates, and plasmids were purified using NucleoSpin 96 Plasmid Core Kit (Macherey-Nagel) and sequenced by the Sanger method (LGC Genomics, Berlin, Germany).

### Preparation of Tn5-mediated GUIDE-seq and AAV-seq libraries

Genomic DNA was isolated from the targeted HSPCs (~2 × 10^5^ to 5 × 10^5^) 10 days for GUIDE-seq and 18 days for AAV-seq after targeting using a GenFind V3 Reagent Kit according to the manufacturer’s protocol (Beckman Coulter). Genomic DNA (100 ng) was fragmented using Tn5 transposase (IIlumina) in 20 μl of reaction at 55°C for 7 min. Tagmented DNA fragments were purified by using Zymo DNA Clean and Concentrator-5 according to the manufacturer’s protocol (Zymo Research). To verify the tagmentation (~0.5 to 1.5 kb), 1/10 Zymo elutes were loaded on 1.2% agarose gel (fig. S8). Preparations of GUIDE-seq and AAV-seq libraries followed previous protocols (*15*, *42*) with specific primer sets (table S3). Last, the GUIDE-seq and AAV-seq libraries were loaded onto Illumina MiniSeq for deep sequencing.

### Preparation of amplicon-seq libraries

To perform amplicon deep sequencing, on- and off-target sites, identified by GUIDE-seq, were amplified from genomic DNA of the targeted HSPCs by PCR using Platinum SuperFi PCR Master Mix (Thermo Fisher Scientific) with gene-specific primers including overhang adapter sequences that are comparable to Illumina Nextera XT index adapters (table S3) and following PCR conditions: 98°C for 2 min, 20 cycles (98°C for 10 s, 60°C for 30 s, 72°C for 30 s), and 72°C for 5 min. PCR products were purified using AMPure XP beads (Beckman Coulter), quantified using a Qubit dsDNA HS assay kit (Invitrogen), and normalized to 1 ng/μl. For multiplexing sequencing libraries, these PCR products were indexed through a second PCR with Nextera XT DNA Library Preparation Kit v2 set A (Illumina) using Platinum SuperFi PCR Master Mix (Thermo Fisher Scientific) and following PCR conditions: 98°C for 2 min, 10 cycles (98°C for 10 s, 60°C for 30 s, 72°C for 30 s), and 72°C for 5 min. Indexed PCR products were cleaned using AMPure XP beads (Beckman Coulter), quantified using a Qubit dsDNA HS assay kit (Invitrogen), normalized to 10 ng/μl, and pooled. The amplicon libraries were loaded onto Illumina MiniSeq for deep sequencing.

### LAM-HTGTS

To detect all potential genome editing outcomes, we modified the LAM-HTGTS method (*12*). In brief, we performed a Linear PCR from 330 ng of genomic DNA (~50,000 genomes) using PrimeSTAR GXL polymerase (Takara) with an external biotinylated primer that anneals to a genomic sequence outside of the 5′ or 3′ HAs that excludes the contamination by the remaining AAV donor vectors (table S3). Linear PCR was performed with the following PCR conditions: 98°C for 5 min, 80 cycles (98°C for 30 s, 65°C for 30 s, 68°C for 90 s), and 68°C for 2 min. Biotinylated PCR products were then captured by Streptavidin Dynabeads MyOne C1 (Life Technologies). Captured products were cleared off the genomic DNA by washing. Products were ligated with a double-stranded adapter harboring a degenerate hexanucleotide overhang and protected from self-ligation by amino C3 linkers on 5′-ends. Illumina adapters were introduced via nested PCR on the bead-captured products. Internal primers annealed in close vicinity to the cleavage sites (~50 to 100 bp) and harbored Illumina Nextera adapter sequences on their 5′-ends. Q5 polymerase [New England Biolabs (NEB)] was used for the nested PCR. The PCR products were purified using ProNex beads (Promega) and quantified using a Qubit dsDNA HS assay kit (Invitrogen). For multiplexing sequencing libraries, these PCR products were indexed through a PCR with Illumina indices using Q5 polymerase (NEB). The LAM-HTGTS libraries were loaded onto Illumina MiSeq or MiniSeq for deep sequencing.

### Computational analysis

The pipelines for analyzing the GUIDE-seq, AAV-seq, and LAM-HTGTS were written on the basis of the built off of code in previous publications (*47*, *48*) and shown in fig. S9. The Scripts and Jupyter notebooks are available (github.com/EricDanner). For GUIDE-seq and AAV-seq, samples were demultiplexed, reads were checked for correct priming, and the sequences of AAV inverted terminal repeats (ITRs) and dsODN were trimmed to adjacent genomic sequences using Cutadapt (github.com/MarcelMartin). The adjacent genomic sequences are globally mapped to human genome reference (hg38) using Bowtie 2 (*49*). Perfect mapped reads are checked for overlap of regions within 5000 bp of predicted off-target sites using CRISPOR (*37*) and CrispRGold (*38*) programs. For LAM-HTGTS, samples were demultiplexed and checked for correct priming, and reads were trimmed to the genome interface. Trimmed sequences were aligned first locally to the AAV ITRs. Reads without alignment to AAV ITRs were mapped on in silico–generated amplicons of expected outcomes, following the previous publication (*47*), using Bowtie 2. Perfect mapped reads are then quantified for HDR count. Reads that cover the sgRNA break site are analyzed and quantified for indel/and deletion and inversion outcomes. To determine nuclease-driven translocations, reads that did not align to this point were trimmed with Cutadapt to the proximal sgRNA site and globally aligned to human genome reference (hg38) using Bowtie 2. Perfectly aligned reads that overlapped within 5000 bp of predicted off-targets sites were recognized as nuclease-driven translocations. Plotting was done with homemade R scripts.

### Statistical analysis

Statistical tests were performed with Prism 8.0 (GraphPad) using the nonparametric Mann-Whitney test or two-way analysis of variance (ANOVA). *P* values are shown as ***P < 0.001; **P < 0.01; *P < 0.05. For each experiment, at least three independent experiments were performed. Data are shown as means ± SD values. The number of biological replicates for each experiment is shown in the figure legends and shown as data points in figures.

## Notes

View/request a protocol for this paper from *Bio-protocol*.
