# Supplementary material for: Precise CRISPR-Cas–mediated gene repair with minimal off-target and unintended on-target mutations in human hematopoietic stem cells
Source: Sci Adv. 2022 Jun 3;8(22):eabm9106. doi: 10.1126/sciadv.abm9106 (PMC9166625; doi:10.1126/sciadv.abm9106)
Supplement: Supplementary file 1 — Figs. S1 to S11 Tables S1 to S3 [file sciadv.abm9106_sm.pdf]

Science Advances
AAAS

Supplementary Materials for

Precise CRISPR-Cas–mediated gene repair with minimal off-target and unintended on-target mutations in human hematopoietic stem cells

Ngoc Tung Tran *et al.*

Corresponding author: Klaus Rajewsky, klaus.rajewsky@mdc-berlin.de;
Van Trung Chu, vantrung.chu@mdc-berlin.de

Sci. Adv. **8**, eabm9106 (2022)
DOI: 10.1126/sciadv.abm9106

This PDF file includes:

Figs. S1 to S11
Tables S1 to S3

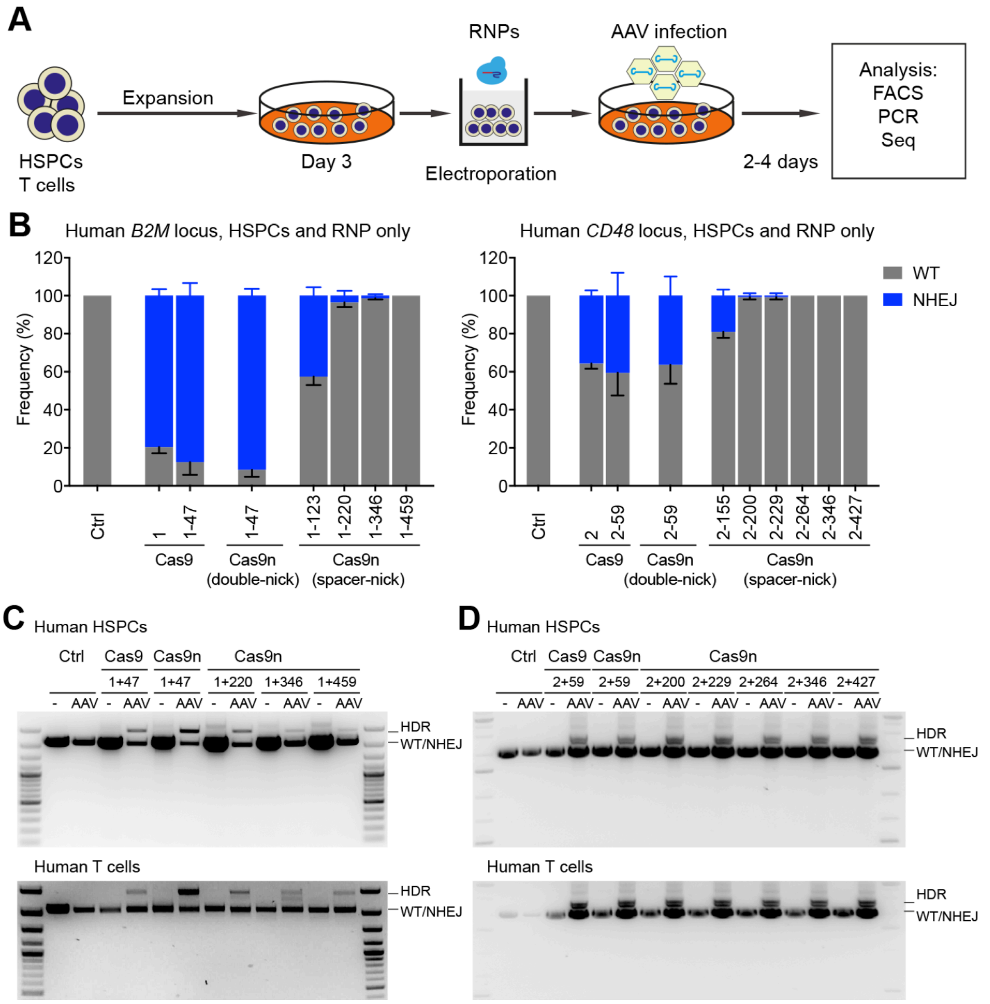

Fig. S1. Gene targeting at the *B2M* and *CD48* loci in human HPSCs and T cells.

(**A**) Experimental scheme of gene insertion in human HSPCs and T cells which were electroporated with RNPs and subsequently infected with AAV donor vectors. (**B**) Frequencies of NHEJ (blue) and wild-type (WT, grey) sequences at the targeted *B2M* (left) and *CD48* (right) loci in human HSPCs treated with control (Ctrl, no RNPs), Cas9, double-nick or spacer-nick RNPs. Data are shown as means ± SD from three independent experiments. Correct integration PCR showing HDR and WT/NHEJ sequences at the targeted *B2M* (**C**) and *CD48* (**D**) loci in human HSPCs (upper) and T cells (below) that were treated as indicated.

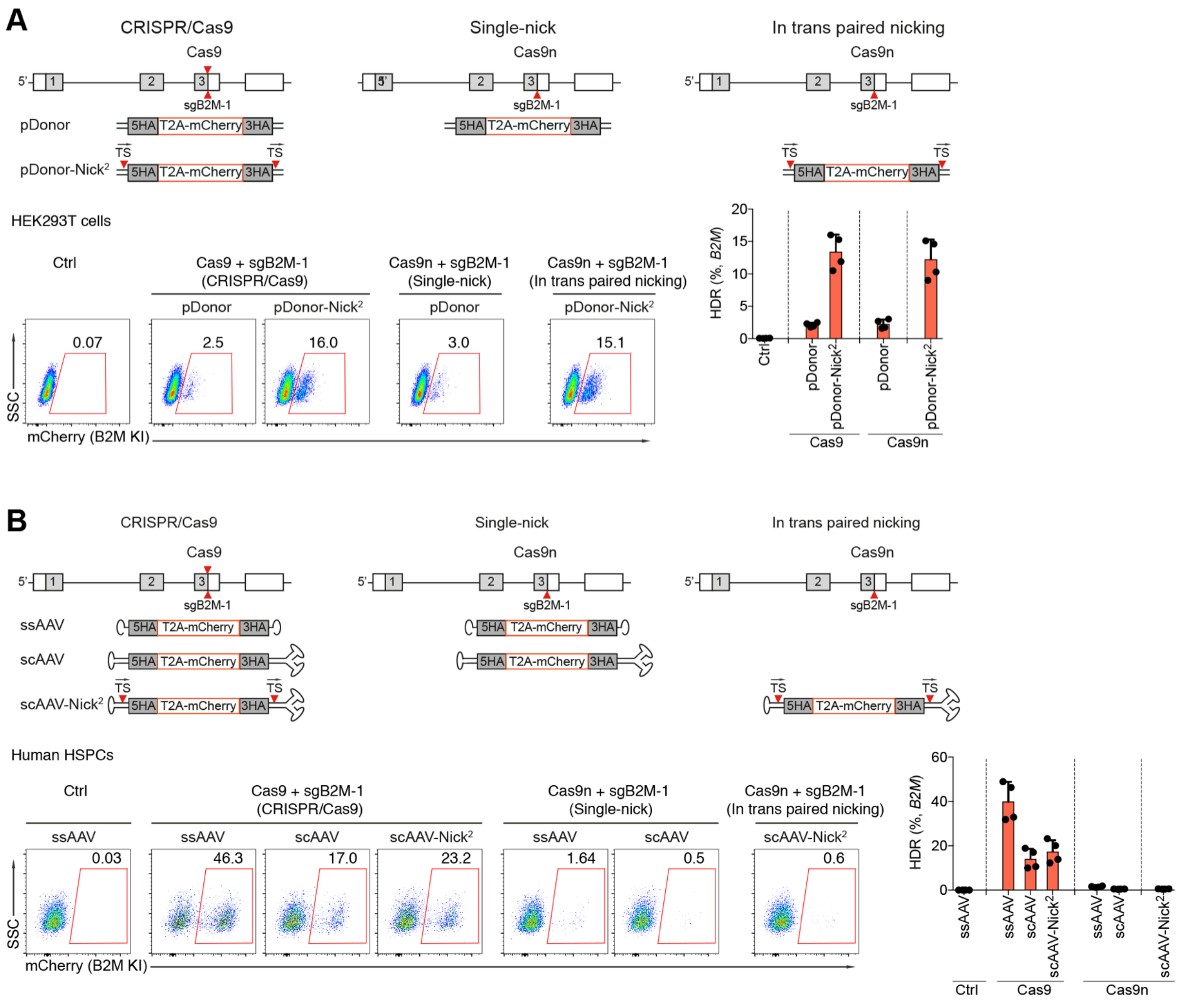

Fig. S2. In trans paired nicking approach leads to inefficient HDR in human HSPCs.

(**A**) Targeting strategy to insert T2A-mCherry into the human *B2M* locus using CRISPR/Cas9, single-nick or in trans paired nicking approach in HEK293T cells. Donor plasmids without (pDonor) or with 2 target sequences (TS) (pDonor-Nick^2) were used. The common sgRNA targeting the *B2M* is indicated in red. FACS analysis of the percentages of mCherry$^+$ HEK293T cells six days post targeting the *B2M* locus with the indicated methods. Graph summarizes frequencies of mCherry$^+$ (HDR) HEK293T cells as measured by FACS. (**B**) Insertion of T2A-mCherry into the human *B2M* locus using CRISPR/Cas9, single-nick or in trans paired nicking approach in human HSPCs. Single-stranded (ss) AAV and self-complementary (sc) AAV without (scAAV) or with 2 TS (scAAV-Nick^2) were used as donor templates. FACS analysis showing frequencies of mCherry$^+$ HSPCs three days post targeting. Bar graph showing HDR (mCherry$^+$) efficiencies. Data are shown as means ± SD from four independent experiments.

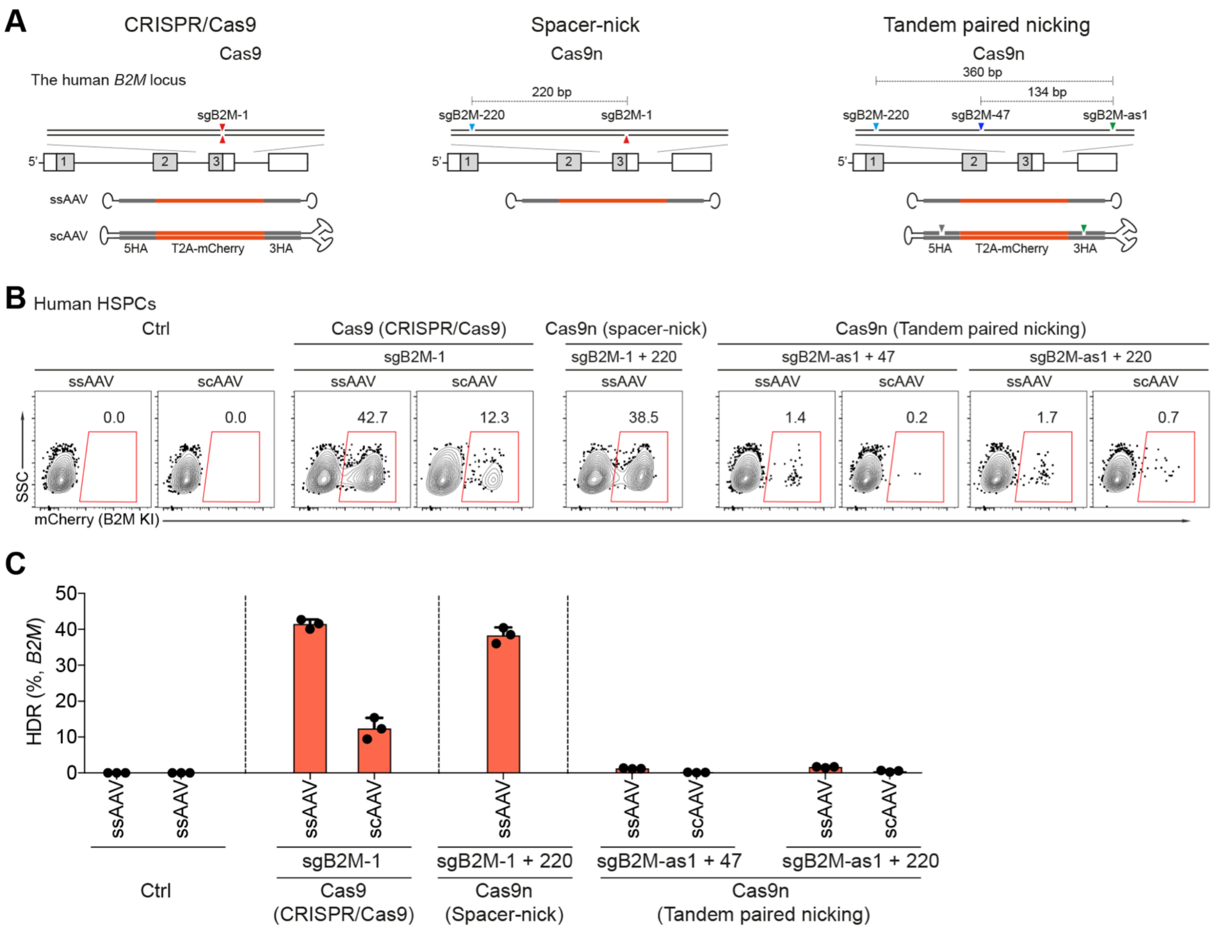

Fig. S3. Tandem paired nicking approach leads to low HDR efficiency in human HSPCs.

(**A**) Experimental scheme to insert T2A-mCherry into the human *B2M* locus in human HSPCs using CRISPR/Cas9, spacer-nick, or tandem paired nicking approach. SsAAV and scAAV donor vectors with the indicated HAs flanking T2A-mCherry fragments were depicted. Common sgB2M-1, sgB2M-47, sgB2M-220, and sgB2M-as1 are indicated as red, dark blue, light blue, and green arrows, respectively. (**B**) FACS analysis of the percentages of mCherry^{+} HSPCs three days post targeting. (**C**) Bar graph showing HDR efficiencies in (**B**). Data are shown as means ± SD from three independent experiments.

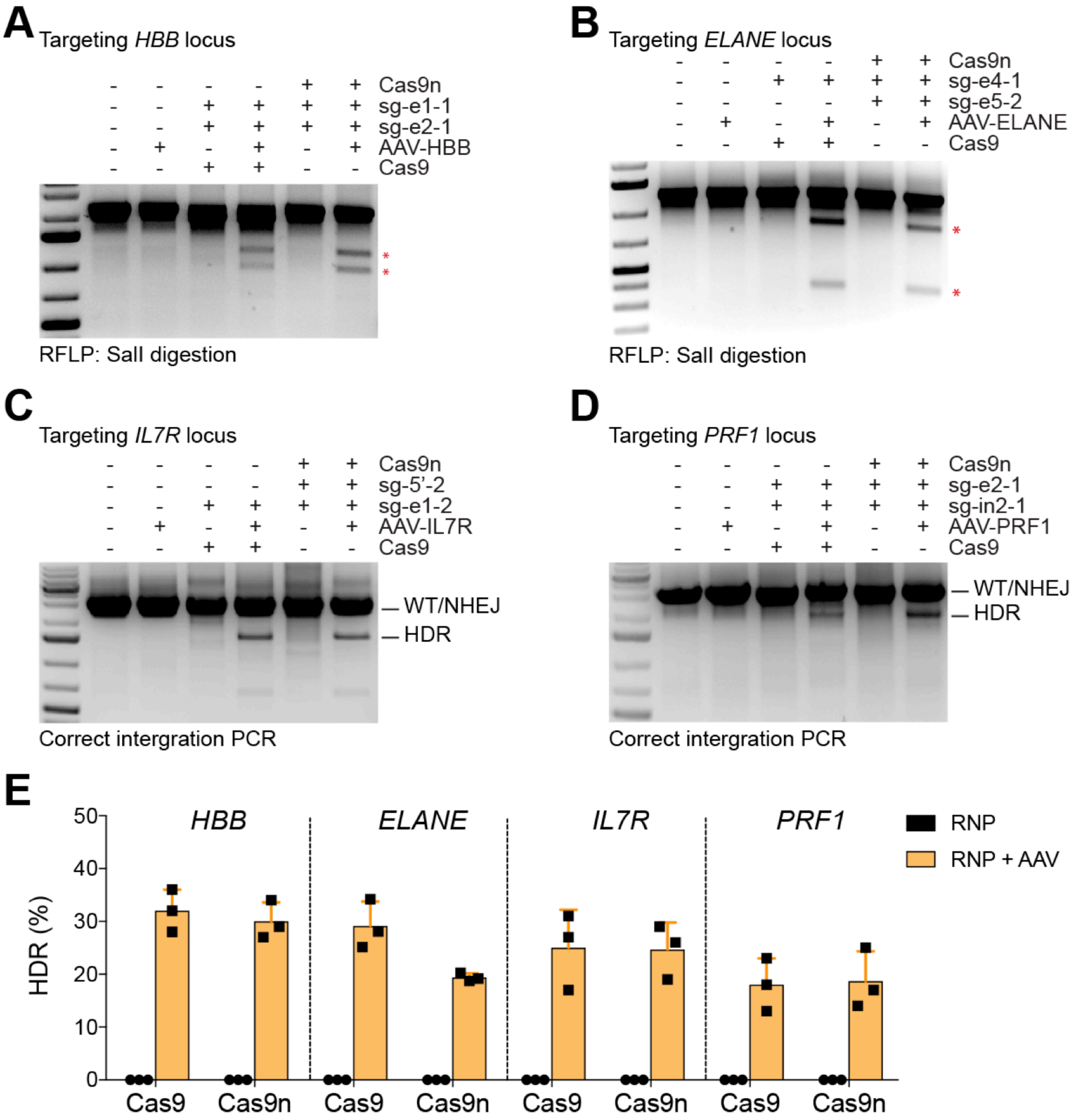

Fig. S4. Gene correction efficiencies in human HSPCs.

SalI-mediated RFLP assays showing the gene correction efficiency of the targeted *HBB* (**A**) and *ELANE* (**B**) loci in the HSPCs treated with indicated approaches. Asterisks depict the SalI-cleaved bands. Correct integration PCR showing WT/NHEJ and HDR events at the targeted *IL7R* (**C**) and *PRF1* (**D**) loci in the HSPCs treated as indicated. (**E**) Quantification of gene correction efficiencies (HDR) at the targeted loci in the HSPCs treated with RNP only (black) or RNP and AAV donor vectors (yellow).

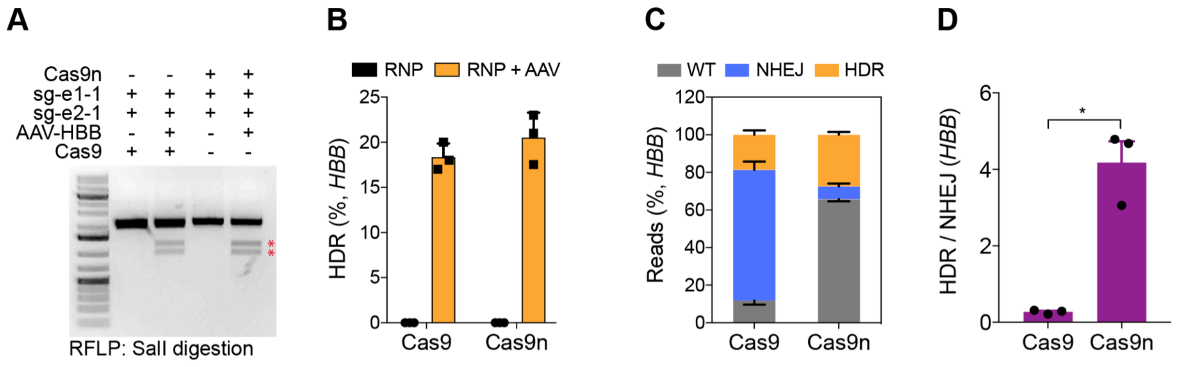

Fig. S5. Spacer-nick leads to efficient *HBB* gene correction in human T cells.

(A) SalI-mediated RFLP assay showing efficiency of *HBB* gene correction in human T cells treated with RNPs only or RNPs and AAV donor vectors. Asterisks indicate the SalI-cleaved bands. (**B**) Quantification of HDR efficiency in T cells shown in (**A**) from three independent experiments. (**C**) Frequencies of WT (grey), NHEJ (blue), and HDR (orange) sequences at the targeted *HBB* locus in human T cells treated as indicated. (**D**) The ratio of HDR:NHEJ at the targeted *HBB* locus. Data represent means ± SD from three independent experiments, * P<0.05.

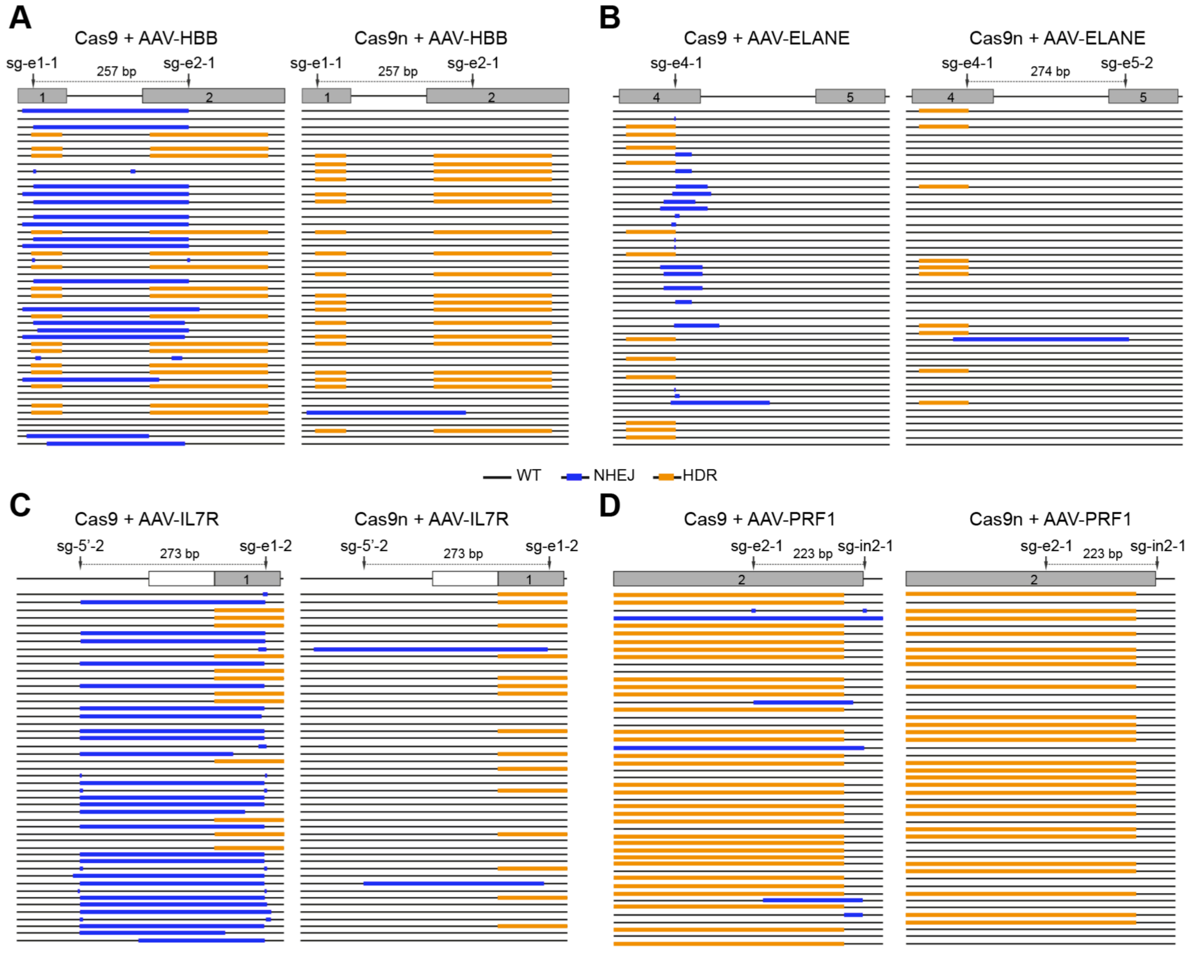

Fig. S6. Sanger sequencing analysis at the targeted loci in human HSPCs.

Representative sequences of WT (black), NHEJ (blue) and HDR (orange) at the targeted *HBB* (**A**), *ELANE* (**B**), *IL7R* (**C**) and *PRF1* (**D**) loci in human HSPCs that received either Cas9 or Cas9n RNPs, and AAV donor vectors.

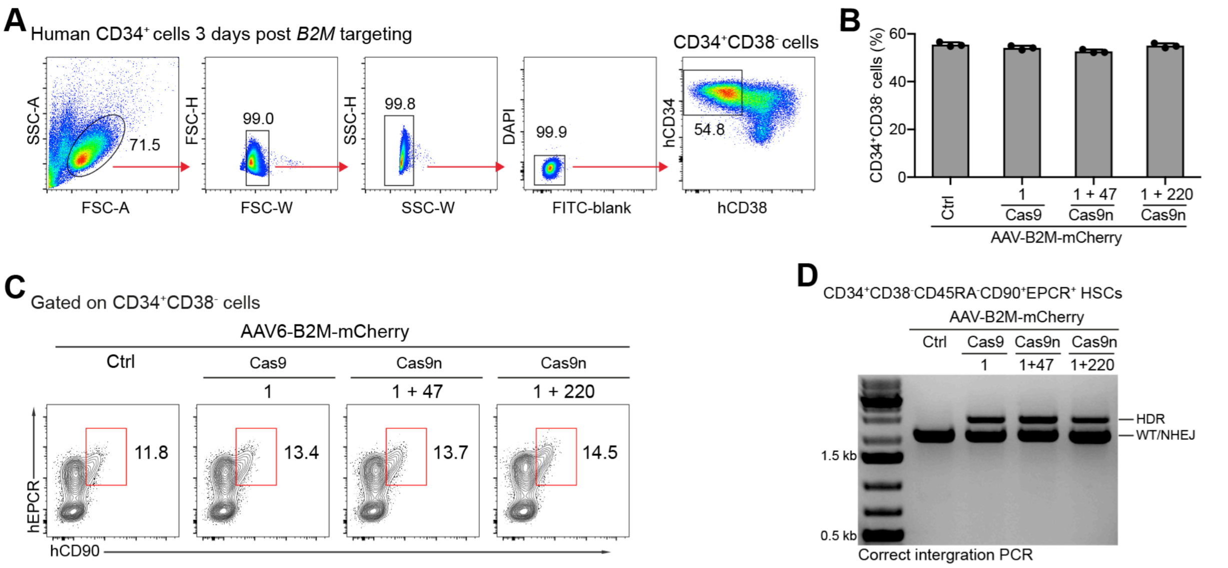

Fig. S7. Gene insertion into the *B2M* locus in long-term HSCs.

(**A**) Gating strategy for CD34$^+$CD38$^-$ population after three days targeting the *B2M* locus in human CD34$^+$ cells. (**B**) Frequencies of CD34$^+$CD38$^-$ HSPCs treated with control (Ctrl, no RNPs), Cas9, double-nick or spacer-nick RNPs, and AAV donor vectors. Data are shown as means ± SD from three independent experiments. (**C**) FACS analysis, pre-gated on CD34$^+$CD38$^-$ cells, showing the co-expression of CD90 and EPCR markers in long-term HSCs. (**D**) Correct integration PCR showing WT/NHEJ and HDR events at the targeted *B2M* locus in the sorted CD34$^+$CD38$^-$CD45RA$^-$CD90$^+$EPCR$^+$ HSCs.

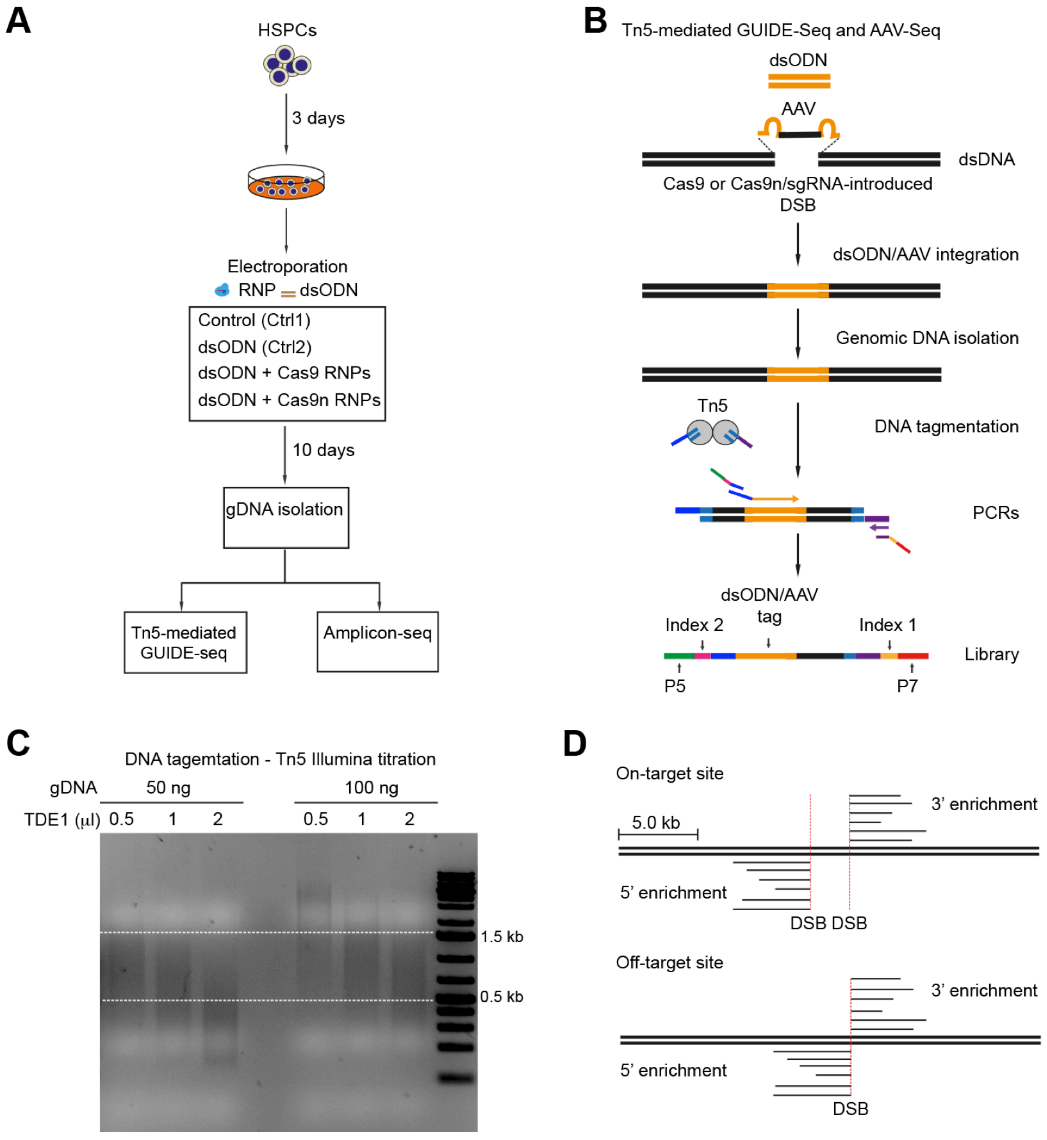

Fig. S8. Nextera Tn5-mediated GUIDE-seq and AAV-seq.

(A) Experimental scheme of GUIDE-seq and Amplicon-seq in the targeted HSPCs. (**B**) Workflow of Tn5-mediated GUIDE-seq and AAV-seq methods. (**C**) Optimization of DNA tagmentation with different amounts of Nextera Tn5 transposase (TDE1) in 20 μl reaction with 50 or 100 ng of genomic DNA. The expected size of tagmented DNA fragments is 0.5-1.5 kb (white lines). (**D**) By mapping of reads to human genome within 5.0 kb distance on both sides (5' and 3'), on- and off-target DSB positions were identified.

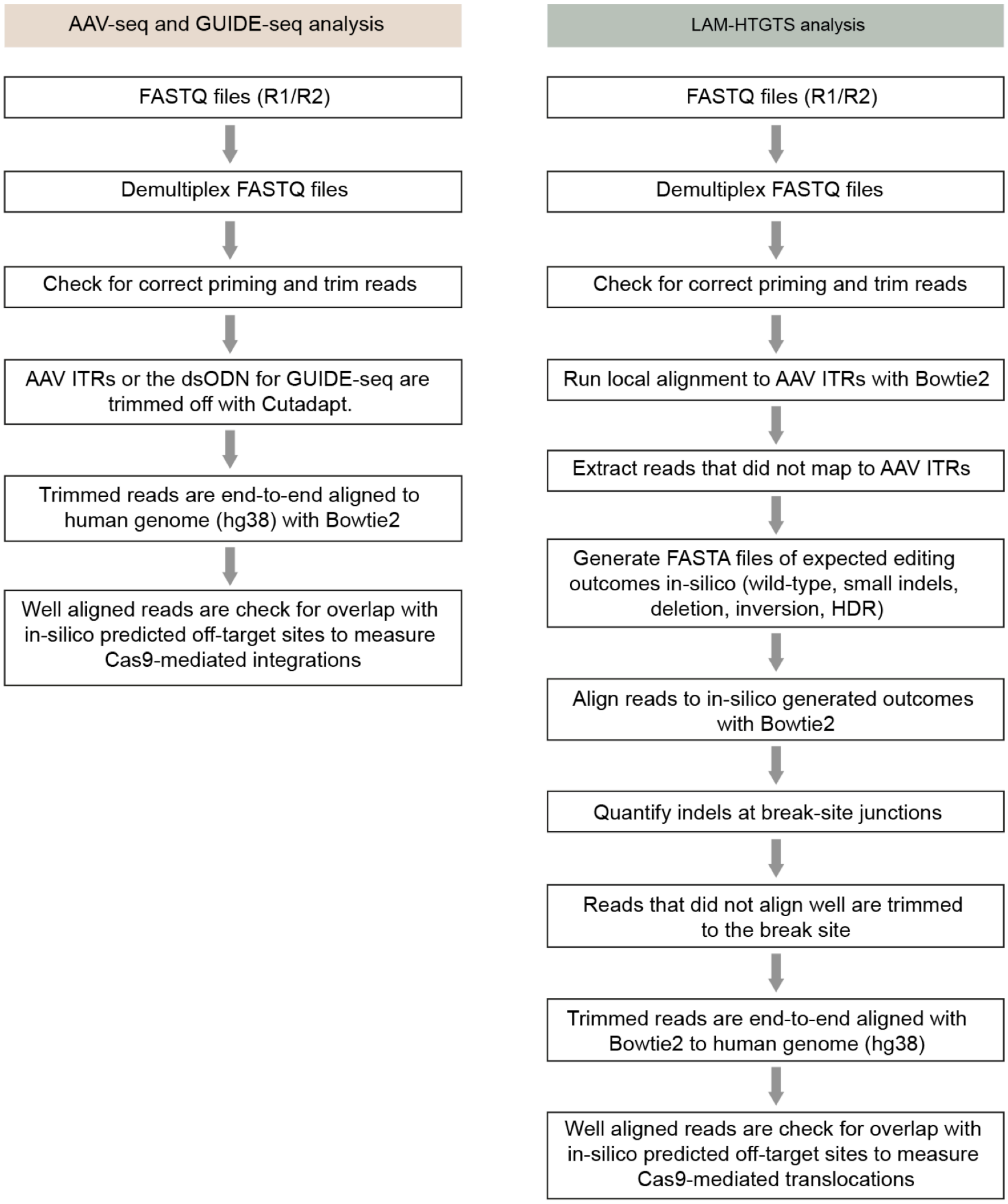

Fig. S9. Pipelines analyze GUIDE-seq, AAV-seq and LAM-HTGTS data.

For GUIDE-seq and AAV-seq analysis, samples were demultiplexed, reads were checked for correct priming and the sequences of AAV inverted terminal repeats (ITRs) and dsODN were trimmed to adjacent genomic sequences that are globally mapped to human genome (hg38) using Bowtie2. Mapped reads that were overlapped the off- predicted target sites, were quantified. For LAM-HTGTS analysis, samples were demultiplexed, checked for correct priming and trimmed to genome interface. The trimmed reads were aligned to sequences of the AAV ITRs for quantifying AAV integrations. The reads that were not aligned to AAV ITRs, were globally aligned to human genome for quantifying wild-type, indel/and deletion, inversion, HDR and translocation events.

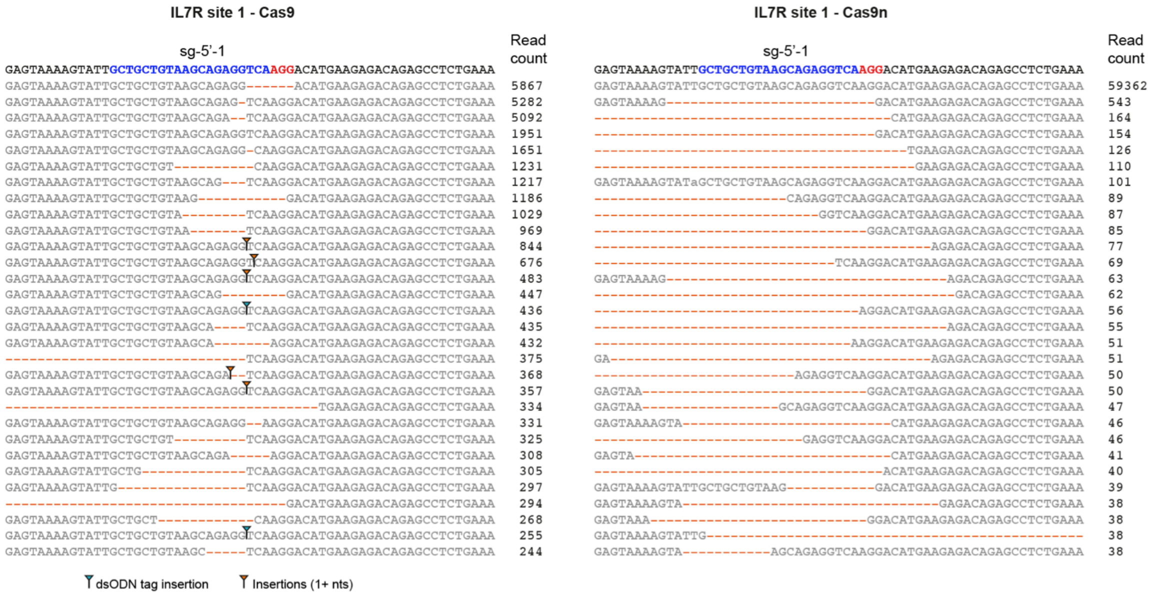

Fig. S10. Amplicon-seq analysis.

On- and off-target sites were amplified from genomic DNA of the targeted HSPCs treated with dsODN tag and either Cas9 or Cas9n RNPs. PCR products were indexed, pooled and sequenced using Miniseq. Indel profiles showing number of intact, deletion/ or insertion (1+ nts) and dsODN tag integration reads at the *IL7R* on-target site 1.

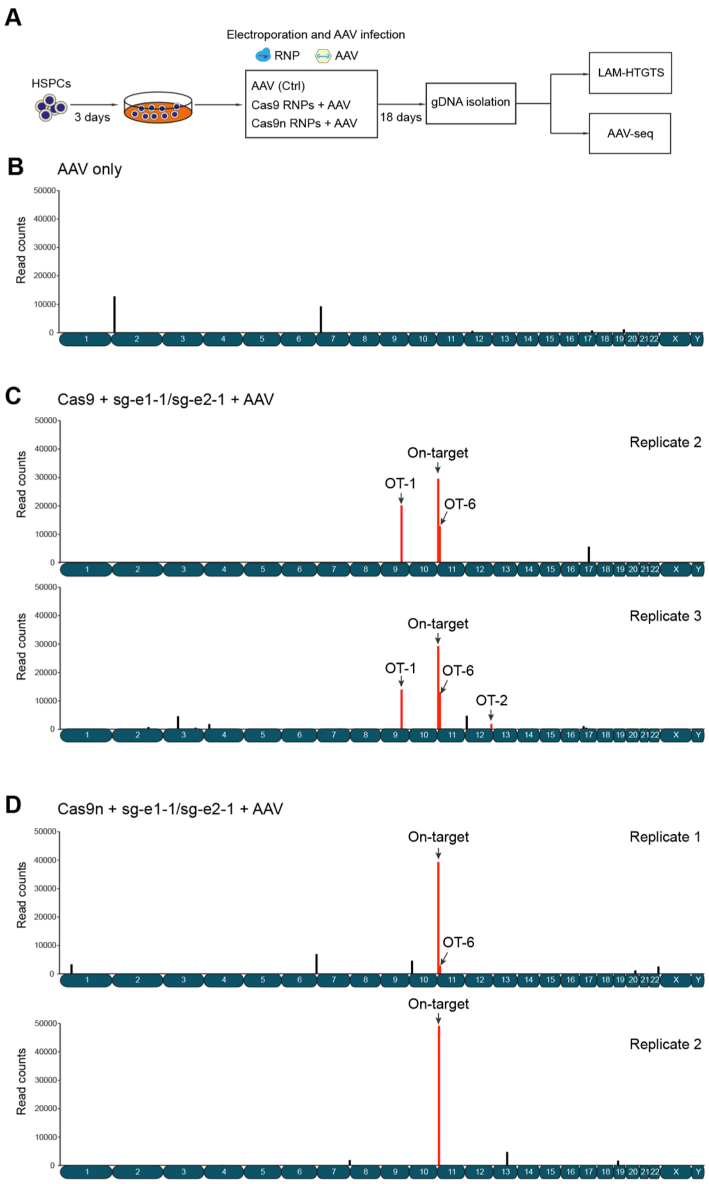

Fig. S11. AAV integration sites.

(**A**) Experimental scheme of AAV-seq and LAM-HTGTS methods. Bar graphs showing number of AAV integration sites mapped on human chromosomes of *HBB*-targeted HSPCs treated with AAV donor vectors only (**B**), sgRNAs/Cas9 (**C**) or sgRNAs/Cas9n RNPs (**D**), and AAV donor vectors. The top hits of AAV integrations are indicated in red including the on-target site and 3 high-risk off-target sites (OT-1, OT-2 and OT-6), identified by GUIDE-seq.

Table S1. List of sgRNAs and gene editing efficiencies.

Name	Sequence (5' to 3')	Gene editing (indels)	
		ICE (%)	T7EI (%)
B2M			
sgB2M-1	GAGACATGTAAGCAGCATCA	92	84
sgB2M-47	AAAGACAGTGGAGAAAAAAA	95	83
sgB2M-123	AGAAATAAGGCTGGCAGAAT	92	80
sgB2M-220	CCAATCCAGCCAGAAAGTAC	90	72
sgB2M-346	AAAAGCTAGAGGAAGCCAGT	94	73
sgB2M-459	CTGTGCATCAGTATCTCAGC	83	69
sgB2M-as1	CAATGTTCTCCACATAGTGA	87	85
CD48			
sgCD48-2	GGCCAGAAGATCTTGCCTGT	90	94.1
sgCD48-59	GCTCATCTCAGGTAAGTAAC	94	73.5
sgCD48-155	CAAGAAAACCCTATATGCTT	87	82.0
sgCD48-200	TTGAGAAGGGGCAAGTGTAC	90	89.0
sgCD48-229	GTGTGGGAGCTCACAGAGCA	94	83.9
sgCD48-264	TCCCTGAATCCCATCCTATT	84	89.7
sgCD48-346	TTAAATATCACTGGCTTACC	86	87.2
sgCD48-427	CTCTGGACATCAACGAGGTA	92	84.6
HBB			
sgHBB-ex1-1	CTTGCCCCACAGGGCAGTAA	(*5*)	
sgHBB-Ex1-2	GTAACGGCAGACTTCTCCTC	(*45*)	
sgHBB-Ex1-3	CACGTTCACCTTGCCCCACA	50	44
sgHBB-Ex1-4	CCTTGATACCAACCTGCCCA	42	35
sgHBB-5'UTR-1	CTCAGGAGTCAGATGCACCA	30	25
sgHBB-5'UTR-2	TGCACCATGGTGTCTGTTTG	10	5
sgHBB-5'UTR-3	AAGCAAATGTAAGCAATAGA	65	52
sgHBB-Ex2-1	TCCACTCCTGATGCTGTTAT	57	50
sgHBB-Ex2-2	TCCCACCCTTAGGCTGCTGG	47	53
sgHBB-Ex2-3	GCTCATGGCAAGAAAGTGCT	26	17
sgHBB-Ex2-4	TGCTGTTATGGGCAACCCTA	29	20
sgHBB-Ex2-5	TATGGGCAACCCTAAGGTGA	59	53
ELANE			
sgELANE-Ex4-1	GAGTGCAGACGTTGCTGCGA	(*44*)	
sgELANE-Ex4-2	GACGTTGCTGCGACGGCAGA	49	37
sgELANE-Ex4-3	GCAGGACGCTGGCGATCCCA	52	30
sgELANE-Ex4-4	CGAAACAGACGCCGGCCTGC	7	ND
sgELANE-Ex4-5	GGCACGTACGAAACAGACGC	5	ND
sgELANE-Ex4-6	CCGTCACGTTGAGCTCCTGC	ND	ND
sgELANE-Ex4-7	GTTGAGCTCCTGCAGGACGC	ND	ND
sgELANE-Ex5-1	GGAATTGCCTCCTTCGTCCG	50	41
sgELANE-Ex5-2	ATTGCCTCCTTCGTCCGGGG	81	72
sgELANE-Ex5-3	ACCTTGTCTGCCTCCACAGG	49	80
sgELANE-Ex5-4	CTGCCTCCACAGGGGGACTC	53	85
sgELANE-Ex5-5	GGCAGCCCCTTGGTCTGCAA	6	10
IL7R			
sgIL7R-5'UTR-1	GCTGCTGTAAGCAGAGGTCA	54	23.0
sgIL7R-5'UTR-2	AGTATTGCTGCTGTAAGCAG	52	43.9
sgIL7R-5'UTR-3	AGAAGGCAAGTTCCAGAAAC	27	22.8

sgIL7R-5'UTR-4	TATAAACACAGCAAAAAAGA	35	27.8
sgIL7R-5'UTR-5	ACTTATCTAACCACAGACAA	58	49.5
sgIL7R-Ex1-1	TCTAGGTACAACTTTTGGCA	10	5.3
sgIL7R-Ex1-2	CAAGTCGTTTCTGGAGAAAG	56	18.0
sgIL7R-Ex1-3	GAAAGTGGCTATGCTCAAAA	55	38.4
PRF1			
sgPRF1-ex2-1	TGGCCCTGGTTACATGGCGC	83	ND
sgPRF1-ex2-2	GCGCTTGCACTCTGAGCGTG	48	ND
sgPRF1-ex2-3	CTGGGAAGGAGCCCGAGCGG	63	ND
sgPRF1-in2-1	CGGTGGAGTGCCGCTTCTAC	80	ND
sgPRF1-in2-2	CCGCTTCTACAGGTGAGAGC	10	ND
sgPRF1-in2-3	TAGGGGTGGGGGGCTGGAAA	7	ND
sgPRF1-in2-4	GAAAAGGCGCGGGAAACTCT	8	ND

Gene editing efficiencies of sgRNAs were measured by ICE analysis and T7EI assay. ND: not determined.

Table S2. Potential combination of spacer-nick sgRNAs for gene correction.

sgRNA-1	sgRNA-2	Distance (bp)	HDR (%)	NHEJ (%)
**Targeting *HBB* locus**				
sgHBB-Ex1-1	sgHBB-Ex2-1	257	35	1.3
sgHBB-Ex1-1	sgHBB-Ex2-4	268	27	0.5
sgHBB-Ex1-1	sgHBB-Ex2-5	274	26	0.8
sgHBB-Ex1-2	sgHBB-Ex2-1	273	27	1.8
sgHBB-Ex1-2	sgHBB-Ex2-4	284	22	1.0
sgHBB-Ex1-2	sgHBB-Ex2-5	290	19	0.7
sgHBB-Ex1-3	sgHBB-Ex2-1	248	23	1.4
sgHBB-Ex1-4	sgHBB-Ex2-1	206	31	4.6
sgHBB-5'UTR-1	sgHBB-Ex2-2	223	10	0.9
sgHBB-5'UTR-2	sgHBB-Ex2-2	236	18	1.3
sgHBB-5'UTR-3	sgHBB-Ex2-2	284	ND	ND
**Targeting *ELANE* locus**				
sgELANE-Ex4-1	sgELANE-Ex5-1	271	28	2.2
sgELANE-Ex4-1	sgELANE-Ex5-2	274	29	1.6
sgELANE-Ex4-1	sgELANE-Ex5-3	210	30	3.5
sgELANE-Ex4-1	sgELANE-Ex5-4	217	29	2.9
sgELANE-Ex4-2	sgELANE-Ex5-1	278	19	1.6
sgELANE-Ex4-2	sgELANE-Ex5-2	281	13	1.0
sgELANE-Ex4-2	sgELANE-Ex5-3	217	17	2.9
sgELANE-Ex4-2	sgELANE-Ex5-4	224	16	2.1
sgELANE-Ex4-3	sgELANE-Ex5-3	267	ND	ND
sgELANE-Ex4-3	sgELANE-Ex5-4	274	ND	ND
**Targeting *IL7R* locus**				
sgIL7R-5'UTR-1	sgIL7R-Ex1-2	279	10.0	1.7
sgIL7R-5'UTR-1	sgIL7R-Ex1-3	294	15	1.2
sgIL7R-5'UTR-2	sgIL7R-Ex1-2	273	20	1.4
sgIL7R-5'UTR-2	sgIL7R-Ex1-3	288	25	2.0
sgIL7R-5'UTR-5	sgIL7R-Ex1-2	194	20	14
sgIL7R-5'UTR-5	sgIL7R-Ex1-3	209	17	5.2
**Targeting *PRF1* locus**				
sgPRF1-ex2-1	sgPRF1-in2-1	223	55	3.1
sgPRF1-ex2-2	sgPRF1-in2-1	452	30	1.0
sgPRF1-ex2-3	sgPRF1-in2-1	367	20	1.3

This table shows additional pairs of spacer-nick sgRNAs for spacer-nick-mediated gene correction. Percentages of HDR and NHEJ sequences were measured by TOPO cloning and Sanger sequencing. ND: not determined.

Table S3. Primers were used in this study.

Primer name	Sequence (5'>>3')
	**Primers for T7EI, RFLP and correct integration PCR**
B2M-T7-For	GATTCAGGTTTACTCACGTCATCCAGCAGA
B2M-T7-Rev	CCATACTGGCAGTTCCTTTGCCCTCTC
B2M-5HAextern-For	CATGTAGACTCTTGAGTGATGTGTTAAGGAATGCTATGA
B2M-3HAextern-Rev	ACTGGGCAGATCATCCACCTTCCTGATGCT
CD48-T7-For	GTGACAGTGTGGTATCTGACCATGAGAACA
CD48-T7-Rev	CTGTCTGACAGATTTGGGACAACTTCAGGA
CD48-5HAextern-For	CCAGCACTCTGGGAAATACCAGAGTCAGAT
CD48-3HAextern-Rev	CGCCTACCTGCATATAGTGGACAACTCTGG
HBB-T7-For	CCAGAAGAGCCAAGGACAGGTACGGCTG
HBB-T7-Rev	CGATCCTGAGACTTCCACACTGATGCAATC
HBB-5HAextern-For	ACTCTTGCAGATTAGTCCAGGCAGAAACAG
HBB-3HAextern-Rev	ATGAACATGATTAGCAAAAGGGCCTAGCTT
ELANE-T7-For	CTCAACGGGTCGGCCACCATCAACGCCA
ELANE-T7-Rev	TGTCCTCGGAGCGTTGGATGATAGAGTC
ELANE-5HAextern-For	CCAGGCTGGAGCGCAGTGGCACAATCTCAG
ELANE-3HA-Rev	CCTCGGAGCGTTGGATGATAGAGTCGATCC
IL7R-T7-For	GCATAGTGGCATTTGCCTGTAGTCCTAGCT
IL7R-T7-Rev	CCATAGTATTGATCCCCTGCCCCCTGTCAT
IL7R-5HAextern-For	CCATGGGAGCAATTCTACCTAATGACAGTGAATCCA
IL7R-3HAextern-Rev	CAACTTTCAACAAGCAGCAACTGAATGTGAATTCCT
IL7R-modicDNA-For	TTAAGTTGCATCATTTTGTCTGACTAGGTGTCCTTC
PRF1-T7-For	TATCTCAGCCCCTCCCCTTC
PRF1-T7-Rev	TGGGACCTCCTGGATGAGAG
PRF1-5HAextern-For	CCACCCTTCAAGTCACACCTTTGGG
PRF1-3HAextern-Rev	GGGGGAGTGTGTACCACATGGAAAc
PRF1-modicDNA-For	ACAGCCACAGATGCCTACGTGAAGC
	**Primers for Amplicon-seq**
ON-HBB-1-For	TCGTCGGCAGCGTCAGATGTGTATAAGAGACAGAGCAGGGAGGGCAGGAGCCAGGGCTGGGCA
On-HBB-1-Rev	GTCTCGTGGGCTCGGAGATGTGTATAAGAGACAGGGTCTCCTTAAACCTGTCTTGTAACCTTGA
OT-1-For	TCGTCGGCAGCGTCAGATGTGTATAAGAGACAGCTTTCCCGTTCTCCACCCAATAGCTATGGA
OT-1-Rev	GTCTCGTGGGCTCGGAGATGTGTATAAGAGACAGGGTACGGCCTAAGAAATTATAGTTTAGCAGT
OT-2-For	TCGTCGGCAGCGTCAGATGTGTATAAGAGACAGCGCTTCCATCTGATCAGGAGGTCTCCAGTA
OT-2-Rev	GTCTCGTGGGCTCGGAGATGTGTATAAGAGACAGTTATCTGTGTCATCCCAGGAAAGCTTGTCT
OT-3-For	TCGTCGGCAGCGTCAGATGTGTATAAGAGACAGCAGTGCTATGGGAAATAATGATTGTTGCTT
OT-3-Rev	GTCTCGTGGGCTCGGAGATGTGTATAAGAGACAGGGAAGCCAGTAACTCTAAGCCCCTGTAATT
ON-HBB-2-For	TCGTCGGCAGCGTCAGATGTGTATAAGAGACAGGGCACTGACTCTCTCTGCCTATTGGTCTAT
ON-HBB-2-Rev	GTCTCGTGGGCTCGGAGATGTGTATAAGAGACAGGGAAAGAAAACATCAAGCGTCCCATAGACT
OT-4-For	TCGTCGGCAGCGTCAGATGTGTATAAGAGACAGCAGAGAAGACTCTTGGGTTTCTGATAGGCA
OT-4-Rev	GTCTCGTGGGCTCGGAGATGTGTATAAGAGACAGGCCAGGCCATCACTAAAGGCACCTAGCACC
ON-ELANE-1-For	TCGTCGGCAGCGTCAGATGTGTATAAGAGACAGCACGGTGCCTGTTGCTGCAGTCCGGGCTGG
ON-ELANE-1-Rev	GTCTCGTGGGCTCGGAGATGTGTATAAGAGACAGCCTTCTGGGCAGGAACCGTGGGATCGCCAG
ON-ELANE-2-For	TCGTCGGCAGCGTCAGATGTGTATAAGAGACAGGGGCCTCGCAGTCCAGCTTCCCCACCTTGT
ON-ELANE-2-Rev	GTCTCGTGGGCTCGGAGATGTGTATAAGAGACAGGGTTGTCCTCGGAGCGTTGGATGATAGAGT
OT-7-For	TCGTCGGCAGCGTCAGATGTGTATAAGAGACAGCCCTGGAGAACGATCGTATTTGGATGGCCT
OT-7-Rev	GTCTCGTGGGCTCGGAGATGTGTATAAGAGACAGGCAGCGATTCCAGCTGGCAGCGCCGTGACA
OT-8-For	TCGTCGGCAGCGTCAGATGTGTATAAGAGACAGCCAACTCCCCTATTGTTGATATTGTCCCTG
OT-8-Rev	GTCTCGTGGGCTCGGAGATGTGTATAAGAGACAGGATGCGAACGACAACGCACCAGCGTTTGCT
ON-IL7R-1-For	TCGTCGGCAGCGTCAGATGTGTATAAGAGACAGAACCACAGACAAGGGAAGTATAAACACAGC
ON-IL7R-1-Rev	GTCTCGTGGGCTCGGAGATGTGTATAAGAGACAGCTGCCTTACTCTGATGTAAGCACAGTAAGTGT
OT-9-For	TCGTCGGCAGCGTCAGATGTGTATAAGAGACAGAAACCCAACATGGTCAGACCTGGACTGAAG
OT-9-Rev	GTCTCGTGGGCTCGGAGATGTGTATAAGAGACAGTCAACTTGCCCTTTTGGCCTCTACCTTGCT
OT-10-For	TCGTCGGCAGCGTCAGATGTGTATAAGAGACAGAGCTCAGCTTTCTAAACCTTACACCAGTAATG
OT-10-Rev	GTCTCGTGGGCTCGGAGATGTGTATAAGAGACAGAGAGCATAAAGCCTGGTAGGCAGAGGGACA
OT-11-For	TCGTCGGCAGCGTCAGATGTGTATAAGAGACAGAGGCAGCTAATCCTCTGTTGTCAGCTTTCA
OT-11-Rev	GTCTCGTGGGCTCGGAGATGTGTATAAGAGACAGGTTTCCTGTGGCTGTGAGGTAATGCCCCAA
OT-12-For	TCGTCGGCAGCGTCAGATGTGTATAAGAGACAGCCCCAGGCCATCAGTCGGGGATTGAATACA
OT-12-Rev	GTCTCGTGGGCTCGGAGATGTGTATAAGAGACAGCCCACAGCAGCCACAGTCATCTCTCCGTCA
OT-13-For	TCGTCGGCAGCGTCAGATGTGTATAAGAGACAGACAGAGCCTGGAGAAATCACAAAATACTTGT
OT-13-Rev	GTCTCGTGGGCTCGGAGATGTGTATAAGAGACAGCTTCTAGGAAACACAGTCCTGTACCAGAGC
ON-IL7R-2-For	TCGTCGGCAGCGTCAGATGTGTATAAGAGACAGTCATTTCATACACACTGGCTCACACATCTAC
ON-IL7R-2-Rev	GTCTCGTGGGCTCGGAGATGTGTATAAGAGACAGAGGGAACTGAATAACCTGAAACCATGCTACA
OT-14-For	TCGTCGGCAGCGTCAGATGTGTATAAGAGACAGGAAAGCCCTTGAGCTTTCAATTTGGGGCA
OT-14-Rev	GTCTCGTGGGCTCGGAGATGTGTATAAGAGACAGCTCCCTTGGCATATATGAGGAGGTAAAGTA
ON-PRF1-1-For	TCGTCGGCAGCGTCAGATGTGTATAAGAGACAGCATTGCTGGTGGGCTTAGGAGTCACGTCCA
ON-PRF1-1-Rev	GTCTCGTGGGCTCGGAGATGTGTATAAGAGACAGCCGACGGCACCTGCACCCTCTGTGAA
OT-15-For	TCGTCGGCAGCGTCAGATGTGTATAAGAGACAGCTGCACTGCCGACCATCCACAGTGGACATT
OT-15-Rev	GTCTCGTGGGCTCGGAGATGTGTATAAGAGACAGCTCCTACTTTATGATTGGATCGGGAGGA
ON-PRF1-2-For	TCGTCGGCAGCGTCAGATGTGTATAAGAGACAGACCAGCAATGTGCATGTGTCTGTGGCCG
ON-PRF1-2-Rev	GTCTCGTGGGCTCGGAGATGTGTATAAGAGACAGCCTCCAAGTTTGATTGGAGGACTCTGCCT
	**Primers for GUIDE-seq**
i5Nextera-3'-GSP (-)	TCGTCGGCAGCGTCAGATGTGTATAAGAGACAGGTTTAATTGAGTTGTCATATGTTAATAACGGT
i5Nextera-5'-GSP (+)	TCGTCGGCAGCGTCAGATGTGTATAAGAGACAGATACCGTTATTAACATATGACAACTCAATTAA

i7Nextera-Rev	GTCTCGTGGGCTCGGAGATGTGTATAAG
	**Primers for AAV-seq**
AAV-+-1	GGATCTCGACGCTCTCCCTGGAGTTGGCCACTCCCTCTCTG
i5Nextera-AAV-+-2	TCGTCGGCAGCGTCAGATGTGTATAAGAGACAGTCTCTGCGCGCTCGCTCG
i7Nextera-Rev	GTCTCGTGGGCTCGGAGATGTGTATAAG
	**Primers for LAM-HTGTS**
HBB-5'extern-For	ACTCTTGCAGATTAGTCCAGGCAGAAACAG
HBB-5'-For-nested	AGCCATCTATTGCTTACATTTGCTTCTGACAC
HBB-3'extern-Rev	ACATGATTAGCAAAAGGGCCTAGCTTGGAC
HBB-3'-Rev-nested	TGTCACAGTGCAGCTCACTCAG
ELANE-5'extern-For	CCAGGCTGGAGCGCAGTGGCACAATCTCAG
ELANE-5'-For-Nested	CTTCTGGGCAGGAACCGTG
ELANE-3'extern-Rev	CTTACTCCAGAGATGCCCAAGAGACTCTGGA
ELANE-3'-Rev-nested	CCTCGGAGCGTTGGATGATAGAGTCGATCC
PRF1-5'extern-For	GGCTCCAGCTATAATGGGGGCTC
PRF1-5'-For-nested	AAGTGCCCCCTGTCTCTGCAGC
PRF1-3'extern-Rev	CCCAGGGGAGTATTTCCCCCATT
PRF1-3'-Rev-nested	TTCCAGGGCTCCTAGACCAC
Adapter-upper	GCGACTATAGGGCACGCGTGGNNNNNN[AmC3]
Adapter-lower	[Phos]CCACGCGTGCCCTATAGTCGC[AmC3]
Adapter primer	GTCTCGTGGGCTCGG AGATGTGTATAAGAGACAG NNNNN
Adapter nested primer	TCGTCGGCAGCGTC AGATGTGTATAAGAGACAG NNNNN
